# Metal-Organic Frameworks and Their Composites Towards Biomedical Applications

**DOI:** 10.3389/fmolb.2021.805228

**Published:** 2021-12-21

**Authors:** Yana Ma, Xianglong Qu, Cui Liu, Qiuran Xu, Kangsheng Tu

**Affiliations:** ^1^ School of Basic Medical Sciences, Xi’an Key Laboratory of Immune Related Diseases, Xi’an Jiaotong University, Xi’an, China; ^2^ Key Laboratory of Environment and Genes Related to Diseases, Xi’an Jiaotong University, Ministry of Education, Xi’an, China; ^3^ Department of Hepatobiliary Surgery, the First Affiliated Hospital of Xi’an Jiaotong University, Xi’an, China; ^4^ Laboratory of Tumor Molecular Diagnosis and Individualized Medicine of Zhejiang Province, Zhejiang Provincial People’s Hospital, Affiliated People’s Hospital, Hangzhou Medical College, Hangzhou, China; ^5^ Research Center of Diagnosis and Treatment Technology for Hepatocellular Carcinoma of Zhejiang Province, Hangzhou, China

**Keywords:** metal–organic frameworks, nanomedicine, therapeutics, imaging, theranostics, sensors

## Abstract

Owing to their unique features, including high cargo loading, biodegradability, and tailorability, metal–organic frameworks (MOFs) and their composites have attracted increasing attention in various fields. In this review, application strategies of MOFs and their composites in nanomedicine with emphasis on their functions are presented, from drug delivery, therapeutic agents for different diseases, and imaging contrast agents to sensor nanoreactors. Applications of MOF derivatives in nanomedicine are also introduced. Besides, we summarize different functionalities related to MOFs, which include targeting strategy, biomimetic modification, responsive moieties, and other functional decorations. Finally, challenges and prospects are highlighted about MOFs in future applications.

## 1 Introduction

Nanomedicine, aiming to solve health and medicine problems using nanomaterials with size at nanometer scales, has become a promising field. Applications of nanomedicine include developing delivery systems, contrast agents, sensors for effective chemotherapy, and diagnostics (Nance, 2019). Nanomaterials play an irreplaceable role in nanomedicine, with various resultant materials, such as organic materials, inorganic nanoparticles, and organic–inorganic hybrids, of which metal–organic frameworks (MOFs) are promising platforms in disease theranostics.

MOFs, also named porous coordination polymer (PCP), are constructed by metal ions or clusters covalently binding to organic linkers, which have attained increasing attention of investigations in various fields, from industry such as energy to medicine for therapeutics. As fascinating materials, MOFs exhibit the following exciting superiorities compared with other current existing nanomaterials: 1) versatile MOFs could be easily obtained through kinds of metal ions and organic linkers. Metal ions commonly applicable include zinc (Zn), iron (Fe), cobalt (Co), and zirconium (Zr), while organic linkers include imidazolates, carboxylates, and phenolates. In addition, as various metals and linkers are available, nontoxic or lower toxic precursors could be chosen for high biocompatible MOFs. 2) High specific surface area and tailorable porosity endow MOFs with high loading efficiency, while the tunable size makes it possible for MOFs to be transported throughout the body through enhanced permeability and retention (EPR) effect. 3) As coordination bonds between precursors are labile, MOFs could be degraded to small molecules and excreted out from the body. 4) MOFs are modifiable through surface chemistry and defect structure, which could extend the function of MOFs such as multi-responsiveness and precise targeting (Wang et al., 2018b). All of these features make MOFs ideal candidates for nanomedicine, such as drug delivery, imaging, and sensor. Moreover, MOF composites fabricated by integrating MOFs with functional entities such as nanoparticles and polymer not only retain the structures and functions of MOFs but also endow additional functions to MOFs, which are more favorable for clinical applications (Zhu and Xu, 2014; Li and Huo, 2015).

**FIGURE 1:**
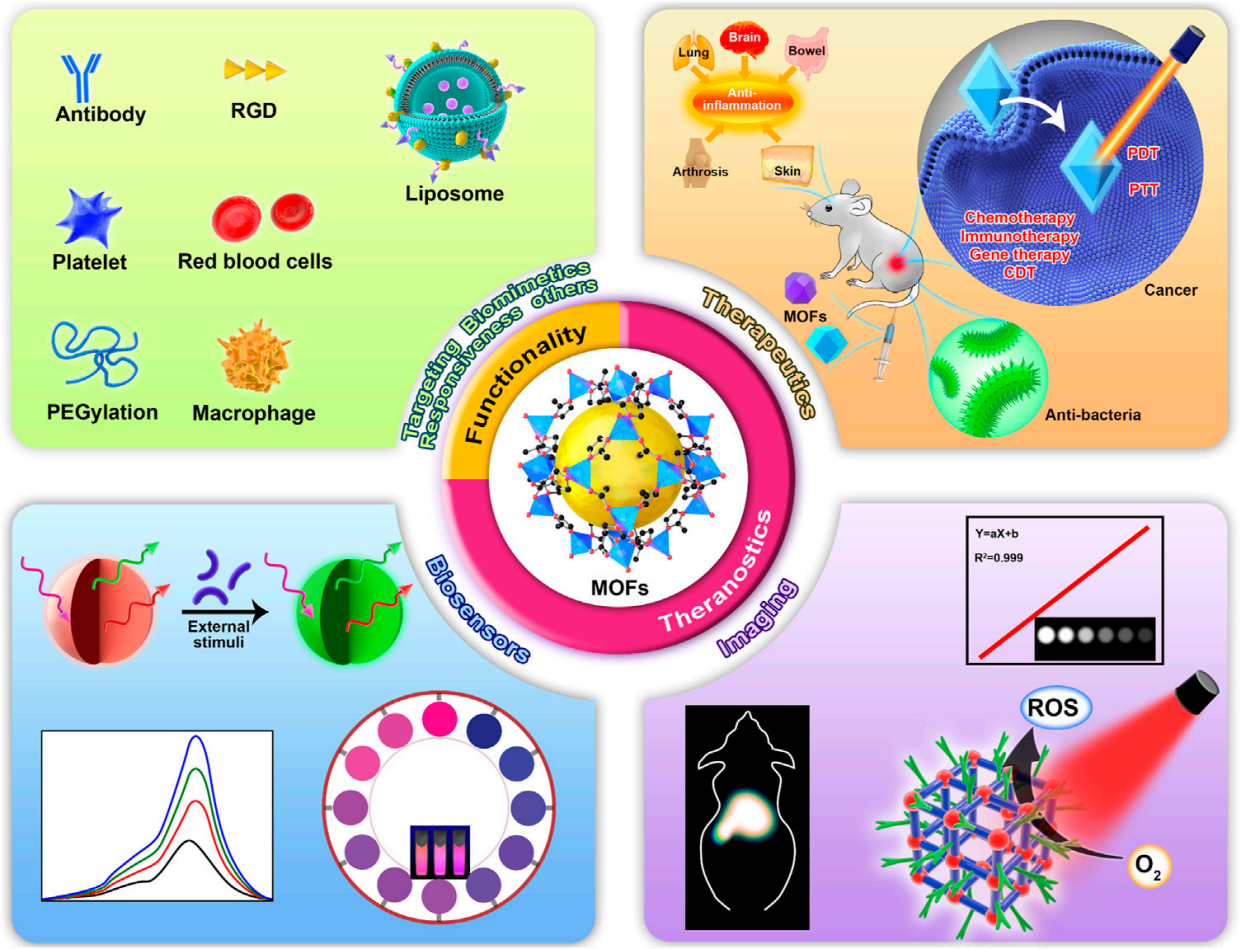
Schematic illustration of functionalities and biomedical applications of metal–organic frameworks (MOFs).

This review aims to summarize the applications of MOFs and their composites in nanomedicine in the last 5 years (Figure 1). Different from various excellent reviews about MOFs, this review mainly emphasizes the strategies/function-orientated therapeutics, imaging, sensors, and theranostics of MOFs and their composites. Also, we outline the kinds and functions of the current surface functionalities of MOFs. In addition, we mention the applications of MOF derivatives. Finally, challenges and perspectives of MOFs and their hybrids in future applications are presented.

## 2 Functionalization

**FIGURE 2:**
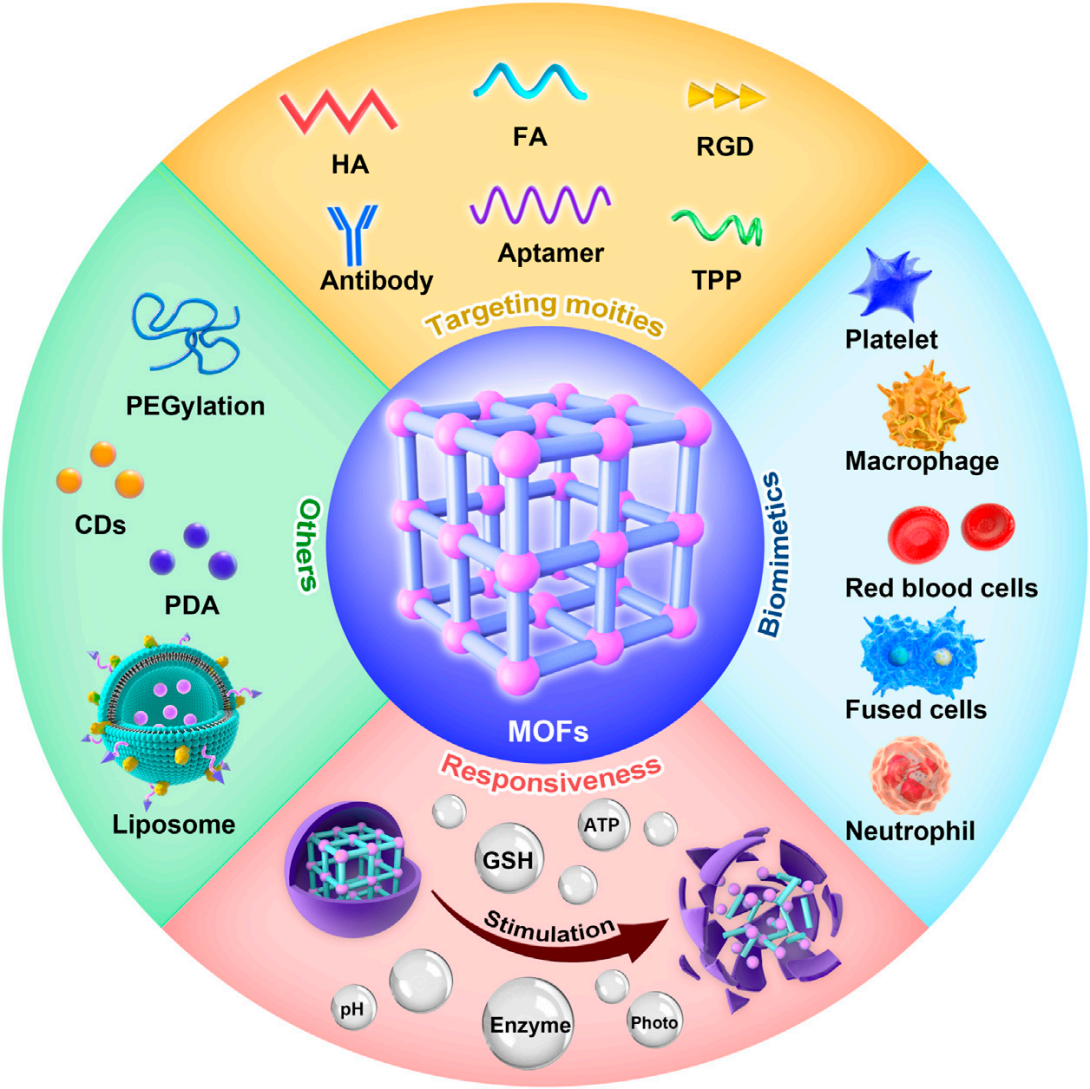
Functionalities of metal–organic frameworks (MOFs), including targeting moieties, biomimetics, and responsiveness.

Functionalization is achieved by adding new functions, features, capabilities, or properties to materials by changing their surface chemistry. Apart from high cargo loading, materials must meet numerous other requirements for precise function *in vivo*. With high biocompatibility, longer circulation time, and the ability to evade the immune system, MOFs can be flushed out from the bloodstream and arrive at the lesion sites through the passive EPR effect. However, drugs that accumulate in lesion sites only through the EPR effect are limited; thus, more effective strategies to improve drug accumulation are needed. By grafting functional targeting entities to the surface of MOFs, they could actively and efficiently get to target sites. In addition, to avoid premature release, it is vital to modify moieties such as PEG to the surface of MOFs. Therefore, this chapter is emphasized on the kinds and applications of surface functionalization of MOFs (Figure 2).

### 2.1 Targeting Modification

Nanomaterials play their roles only when they reach the lesion sites through either passive targeting or active targeting. Conventionally, nanoparticles rely on the passive EPR effect to arrive at the target sites. However, drug accumulation by this way often has unsatisfactory therapeutic effects, with active targeting needed. Active targeting could be mediated by ligand, aptamer, antibody, etc., with all of them depending on the interaction between targeting moieties and specific receptors of target sites.

#### 2.1.1 Hyaluronic Acid-Mediated Targeting

Among various targeting ligands, hyaluronic acid (HA) is a widely used ligand for tumor targeting. HA, a negative linear polysaccharide that exists in the extracellular matrix (ECM), could be recognized by cluster determinant 44 (CD44) overexpressed on the surface of most cancer cells. With excellent biocompatibility and high biodegradability, it has promising applications in nanomedicine (Swierczewska et al., 2016). Inspired by this, various HA-coated MOFs were established for effective targeting through the interaction between HA and MOFs. For example, Sun et al. fabricated a HA/α-TOS@ZIF-8 nanosystem, in which the shell of HA was both an intelligent “switch” and a tumor guider for nanoparticle accumulation in tumor sites via CD44-mediated pathway. Compared with nanoparticles without HA coating, stronger fluorescence (FL) intensity in cells was observed after treatment with HA/α-TOS@ZIF-8 NPs, indicating that HA coating increased the internalization of NPs by cancer cells. Notably, in the tumor microenvironment, HA could be disintegrated by hyaluronidase (HAase), and thus, the MOFs were exposed, and the drugs were further released (Sun et al., 2019). Apart from tumor regions, HA coating could target inflammation sites and also improve the water stability of MOFs. Significantly, HA on the surface of MOFs also alleviates inflammation by reducing the expression of cytokines (Xiong et al., 2020).

#### 2.1.2 Folic Acid-Mediated Targeting

Many cancer cells, including breast, cervical, colorectal, ovarian cancer cells, express a high level of folate receptors (FRs) on their cell surface, and folic acid (FA) is a universally studied targeting agent. As moiety provides ease of modification, FA could bind to various nano-MOFs through interaction between carboxylic groups of FA and positive moieties, such as Hf_6_ cluster of Hf-Mn-NMOF (Bao et al., 2020), amino groups of Uio-66-NH_2_, and IRMOF-3 (Yang et al., 2017a; Gao et al., 2018b). Besides, FA could also be grafted to MOFs through intermediates, exemplified by binding FA to PEI on the surface of gadolinium-porphyrin MOFs (FA-NPMOFs) (Chen et al., 2019b).

#### 2.1.3 RGD Peptide-Mediated Targeting

Integrin family, as adhesion molecules, is involved in mediating the interaction of cell–cell and cell–ECM and regulates the majority of cell processes including cell proliferation and migration, which were found to be pathophysiologically activated in many cell types of diseases. Arg-Gly-Asp (RGD) tripeptide motif, highly expressed in some tumor cells, has a high affinity to adhesion molecules and integrin α_V_β_3_ (Dong et al., 2017b) and thus plays essential roles in drug delivery for cancer treatment. Studies have demonstrated that RGD-modified ZIF-8 could target and treat cancer cells (Dong et al., 2019). Other RGD-containing peptides, such as iRGD (amino acid sequence: CRGDK/RGPD/EC), could also target cancer cells through binding to neuropilin-1 (NRP-1) receptors expressed in tumor and vascular tissues (Liu et al., 2021b).

#### 2.1.4 Aptamer-Mediated Targeting

Aptamers are single-stranded DNA or RNA molecules with high specificity and affinity to their targets. AS1411, a 26-mer DNA aptamer with guanine-rich DNA segment known as G-quadruplex structure, could bind to nucleolin highly expressed both in the cell and on the cell surface (Yazdian-Robati et al., 2020) and thus has attracted increasing interest in targeting treatment. AS1411 has been successfully introduced to various MOFs or their composites, such as ZIF-8 (Dong et al., 2018), γ-CD-MOF (Jia et al., 2019), and FeTCPP/Fe_2_O_3_ MOFs (Zhao et al., 2020b). Furthermore, to further enhance the targeting efficiency, in an experiment for targeting VEGF-overexpressed cancer cells, Chen et al. constructed a doxorubicin (DOX)-loaded Zr^4+^-MOF with dual aptamer, VEGF aptamer, and AS1411 aptamer. On the one hand, this nanoparticle could target cancer cells through AS1411 aptamer; on the other hand, in the presence of VEGF, the MOF could be unlocked and the DOX was released, thus accurately killing the tumor (Chen et al., 2018d).

#### 2.1.5 Other Targeting Functionalities

The antibody is a universal and powerful tool for creating specific cell targeting through antigen–antibody interaction. Cherkasov et al. engineered an antibody-directed magnetic core–shell MOF through covalently binding anti-HER2 antibody to functional polymer on the surface of MOF, which could target and kill HER2/neu-positive cancer cells (Cherkasov et al., 2020). In addition, primary amine-containing MOF could also conjugate to an antibody, which could be used for antibody-targeting strategy. For example, Eu-2-amino-BDC material with an available primary amine in its organic linker could bind to EpCAM antibody, which guided MOFs to human epithelial cell A549 for imaging (Butler et al., 2020).

Mitochondria, one of the essential organelles for various cell functions and energy production, is a promising therapeutic target (Cho et al., 2020). Of all target moieties (Guzman-Villanueva and Weissig, 2017), triphenylphosphine (TPP) is most commonly applicable in MOF nanomedicine from simple Zr-MOF (Zhou et al., 2018a) to composites synthesized by hybridization of porphyrin MOF with UCNPs (Liu et al., 2020).

The molecular marker is another potentially promising targeting approach. For example, hyperphosphorylated tau, as the main feature of Alzheimer’s disease (AD), could be used as a potential target. Immobilized targeting reagent 5-amino-3-(pyrrolo[2,3-*c*]pyridin-1-yl)isoquinoline (defluorinated MK6240, DMK6240) to the surface of MOF Fe-MIL-88B-NH_2_-NOTA-DMK6240/MB could be precisely located in the lesion sites by targeting hyperphosphorylated tau specifically, which then ameliorated AD symptom through inhibiting the aggregation status of hyperphorylated tau (Zhao et al., 2020a).

### 2.2 Biomimetic Modification

Various cell membranes, with their superiority, such as long circulation time and homotypic targeting, have gained increasing attention in bioinspired camouflage strategy. Membrane coatings on MOFs endow them with the superiority of cell membrane. And applicable cell membranes include erythrocyte membrane, platelet membrane, white blood cell membrane, cancer cell membrane, and hybrid cell membrane.

#### 2.2.1 Erythrocyte Membrane/Red Blood Cell Membrane

**FIGURE 3:**
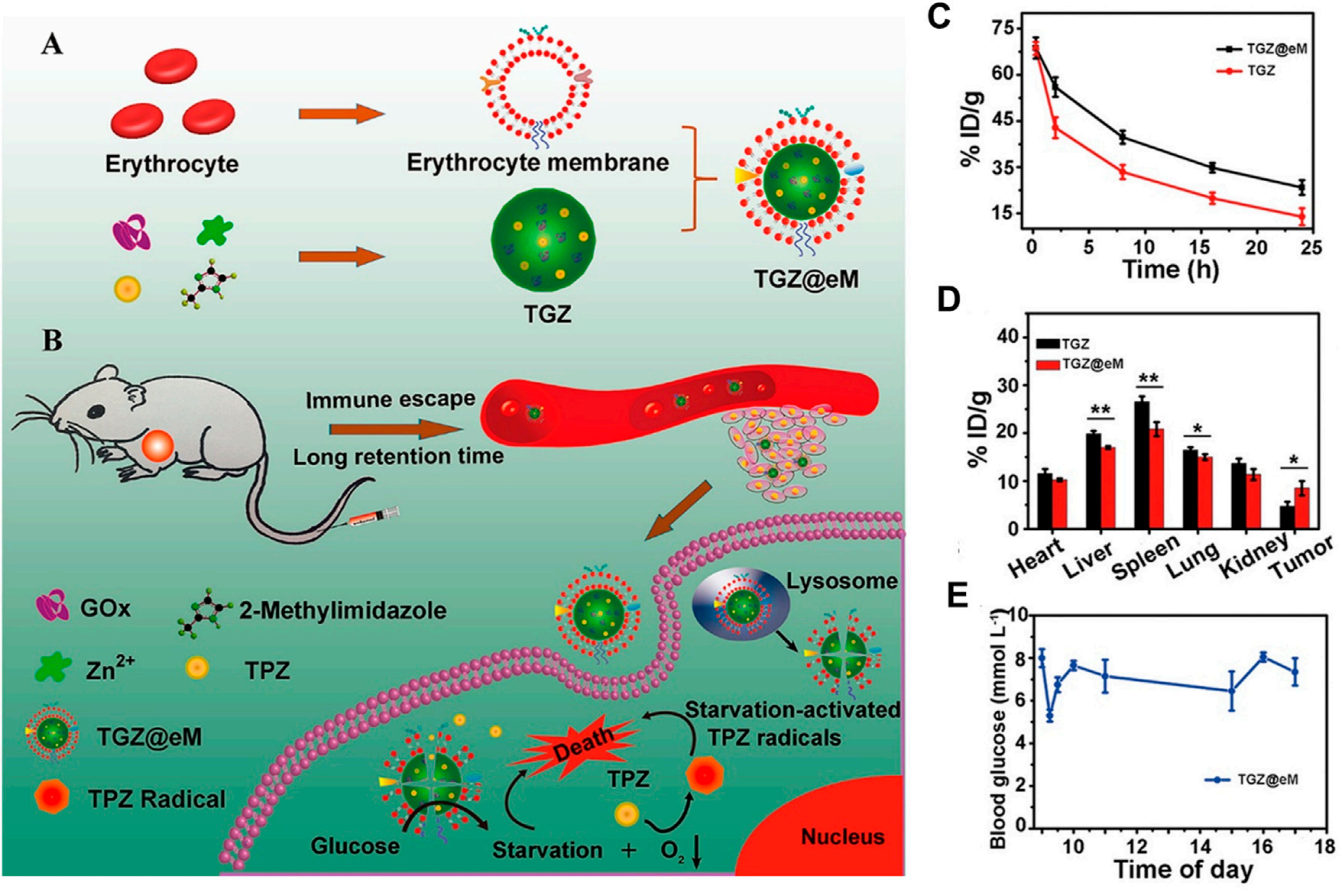
Synthesis, application, and pharmacokinetics of erythrocyte membrane-cloaked metal–organic frameworks (MOF), TGZ@eM. **(A)** Schematic illustration of TGZ@eM synthesis. **(B)** Application diagram of TGZ@eM in cancer treatment. **(C)** *In vivo* pharmacokinetics of TGZ@eM and TGZ. **(D)** Biodistribution of TGZ@eM and TGZ. **(E)** Blood glucose level of mice during daytime after injection with TGZ@eM. Reproduced with permission. Copyright 2018, *ACS Nano*.

Red blood cell (RBC) membranes are the first and widely used cell membranes in nanomedicine, as they have a long life span of about 120 days. In addition, with self-marker CD47 and a series of complement regulatory proteins, RBCs exhibit a strong ability of immune evasion. Inspired by these specialties, Zhang et al. fabricated TGZ@eM nanoreactor with erythrocyte membrane coated on ZIF-8 for co-delivery of glucose oxidase (GOx) and prodrug tirapazamine (TPZ). *In vivo* pharmacokinetics results showed the half-time of TGZ@eM (t_1/2_ = 4.7) was two times longer than that of TGZ (t_1/2_ = 2.4), which enhanced the accumulation of TGZ@eM to the tumor. In addition, *in vitro* cell uptake experiments demonstrated that only a few Rhm B-GOx-ZIF modified with erythrocyte membrane could be taken up by RAW264.7 cells, indicating a better immune escape ability. Owing to these properties of more prolonged blood circulation, better immune evasion, and better tumor targeting, this TCZ@eM exerted better performance for killing tumors (Zhang et al., 2018b) (Figure 3). Similar effects were conducted in O_2_ and ICG-loaded RBC-UiO-66 NPs, wherein RBC membrane-derived O_2_ @UiO-66@ ICG@RBC nanoparticles accumulated in tumor site through their longer circulation ability and immunity-evading superiority and then enhanced the effect of photodynamic therapy (PDT) (Gao et al., 2018a). However, single RBC membrane coating still showed not enough targeting ability; thus, various targeting ligands were integrated into the cell membrane to improve targeting specialty. For example, by modifying erythrocyte membrane with tumor-targeting peptide cyclic RGD (cRGD) and then decorating them onto DOX-loaded ZIF-8, the resultant DOX @ZIF-8@eM-cRGD exhibited both longer blood retention time and specific tumor targeting, which thus improved the tumor inhibition (Lin et al., 2020).

#### 2.2.2 Platelet Membrane

Platelets, fragments released from megakaryocytes, play critical roles in hemostasis. After trauma, platelets could quickly adhere to the injured vascular and trigger the process of coagulation. Platelet membranes exhibit similar circulation properties with RBC; they could also actively target the tumor and inflammation sites through CD44 receptors. Therefore, MOFs coated with platelet membrane might not only possess immunomodulatory ability and capability of selective adhesion to lesion sites but also avoid macrophage uptake (Hu et al., 2015; Fang et al., 2018). This was demonstrated by Zhuang et al. through cloaking platelet membrane onto siRNA-encapsulated ZIF-8. *In vitro* cell uptake experiment showed that platelet membrane decorating endowed nanoparticles with immune evasion ability with less uptake by macrophage. In addition, the targeting ability of SK-BR-3 cells was also improved. Accordingly, the tumor localization was demonstrated in both *in vivo* and *ex vivo* experiments (Zhuang et al., 2020).

#### 2.2.3 White Blood Cell Membrane

White blood cells consist of neutrophils, macrophages, dendritic cells (DCs), and so on. As a crucial component for the body’s defense system, white cell membranes possess various membrane receptors and specific targeting moieties for targeting lesion sites including inflammation and tumor sites.

Macrophages, as innate immune cells, apart from the extended circulation property, could target lesion sites such as tumors, inflammation, and injured vasculatures. Based on these, Chen et al. designed a persistent luminescence nanoparticle@MOF-derived mesoporous carbon core–shell nanocomposite coating by macrophage membrane (MPLMC). The cloaking ability of macrophages was evaluated, and results showed that MPLMC exhibited weaker luminescence than PLMC and PLMC-PEG without membrane coating. In addition, both *in vivo* and *ex vivo* experiments demonstrated the stronger targeting ability for tumors after membrane cloaking (Chen et al., 2020).

Neutrophils, the first white blood cells recruited into lesion sites after trauma, have the natural ability to target inflammation/tumor sites through the transmigration process. By the inflammation-targeting ability of neutrophils, Zhang’s group designed a neutrophil membrane biomimetic nanoplatform. In this nanoplatform, neutrophil membranes were cloaked on a porphyrinic porous coordination network (PCN) decorated by silver nanoparticles (AgNPs). *In vivo* imaging results demonstrated that this nanoparticle was increasingly accumulated at the tumor site with maximum accumulation at 24 h after intravenous administration (Zhang et al., 2020a).

#### 2.2.4 Cancer Cell Membrane

Cancer cell membranes can escape the supervision of the immune system and bind to tumor cells through homotypic adhesion, which made them well-suited and widely used in tumor-targeting nanomedicine. Li et al. developed a cancer cell membrane cloaked cascade bioreactor (mCGP) for synergistic treatment of cancer. In this bioreactor, GOx and catalase (CAT) were embedded into PCN-224, followed by cancer cell membrane coating. After incubation with nanoparticles, 4T1 cells exhibited 2.8-fold stronger FL intensity than African green monkey kidney (COS7) normal cells and other cancer cells. Similar results were conducted in tumor accumulation *in vivo*. Moreover, 2.5-fold weaker FL was observed after treating RAW264.7 cells with mCGP than that of PCN-224. All these results demonstrated that cancer cell membrane coating not only endowed nanoparticles with immune evasion ability but also enhanced the tumor-targeting ability (Li et al., 2017). An et al. combined this superiority of cancer cell membrane with dual-responsive multimodal nanoformulation to realize the gas-sonodynamic combined treatment (An et al., 2020b). More interestingly, incubating different cancer cell membranes with MOF-loaded CRISPR/Cas9 could deliver the CRISPR/Cas9 system to specific cells to realize the personalized gene knockout (Alyami et al., 2020), which is a promising direction for disease treatment.

#### 2.2.5 Hybrid Cell Membrane

Although cell membrane biomimetics owns a tremendous advantage for nanomedicine, single-cell membrane coating could not reach optimal strength owing to their limitations, which could be offset by hybrid cell membranes. Hybrid cell membranes, fused by two or more different cell lines, inherit the properties of dual or multiple cells and thus extend their functions (Liu et al., 2019d). By combination fusion cell membrane acquired from cancer cells and DCs with photosensitizer (PS)-containing MOFs, Zhang’s group engineered an expandable immunotherapeutic nanoplatform (PCN@FM). This PCN@FM nanoparticle could target tumor sites both *in vitro* and *in vivo*. In addition, owing to the expression of cancer antigens and immunological co-stimulatory molecules, PCN@FM not only inhibited rebound of the primary tumor but also restrained distant tumors (Liu et al., 2019d).

### 2.3 Stimulus Responsiveness

Apart from biocompatibility and specific targeting, the controlled release of drugs is significant for optimal therapeutics. Therefore, establishing responsive nanomaterials to realize on-demand release is imperative. With advantages of diversity, flexible structure, and ease of modification, MOFs are such well-suited materials that made drug release possible at desired sites, which could respond to not only disease microenvironment, such as lower pH and higher glutathione (GSH), but also external conditions such as magnetic fields.

#### 2.3.1 pH Responsiveness

**FIGURE 4:**
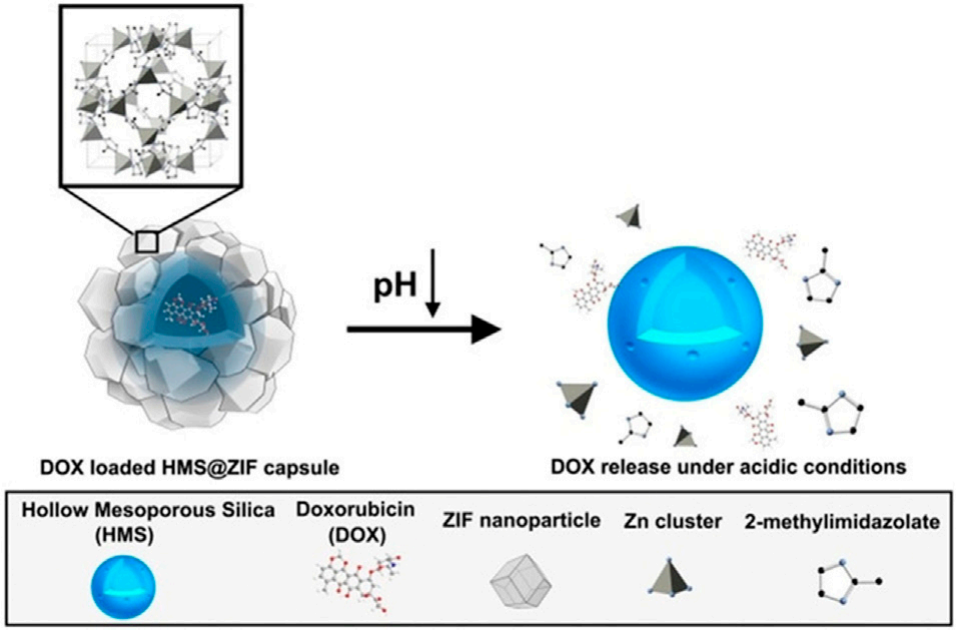
Schematic illustration of hollow mesoporous silica/metal–organic framework (HMS/MOF) pH responsiveness. Reproduced with permission. Copyright 2018, *ChemMedChem*.

pH-responsive MOFs are widely investigated in nanomedicine due to the acidic conditions of lysosomes, tumors, and inflammation. MOFs with intrinsic acidic responsiveness include Zn-MOF, Fe-MOF, and Zr-MOF, which have been widely used in nanomedicine. ZIF-8 in the Zn-MOF family assembled by Zn^2+^ and 2-methylimidazole showed excellent pH sensitivity, which could be disrupted at low pH (5.0–6.0) (Dong et al., 2018; Liang et al., 2018; Dong et al., 2019). For instance, in the drug-release experiment of camptothecin@ZIF-8@RGD, there was almost no drug release under pH 7.4, while about 75% of drugs were released after 24 h in pH 5.0 (Dong et al., 2019). Moreover, composites fusing ZIF-8 with other functional moieties such as polyacrylic acid (PAA) (Yan et al., 2017; Chen et al., 2019a) and hollow mesoporous silica (HMS) (Jia et al., 2018) would not change the pH sensitivity of ZIF-8. Taking DOX-loaded HMS@ZIF-8 hybrid materials as an example, less than 3%, 46.6%, 71.4%, and 85% of DOX were released within 10 h in pH 7.4, 6.0, 5.0, and 4.0, respectively (Jia et al., 2018) (Figure 4). In addition, by incorporating PAA into MOFs, higher pH responsiveness and better drug loading were achieved. ZIF-90 assembly of Zn^2+^ and imidazole-2-carboxaldehyde (IcaH) (Guan et al., 2018), IRMOF-3 composed of Zn^2+^ ions and 2-aminoterephthalic acid (Liu et al., 2019c) were also pH driven. In addition, Fe-MOFs also exhibit pH sensitivity, and discharge of drug in drug-loaded MIL-100 and MIL-101(Fe) (Cabrera-Garcia et al., 2019; Gupta et al., 2019) was observed at pH 5, which boosted the intracellular release of drugs. Zr-porphyrin MOF(PCN-222) also showed pH-responsive features with a cumulative release rate of 86.29% at pH 5.5 compared with 63.23% at pH 7.2 (Leng et al., 2018).

To those with no inherent pH sensitivity, immobilized functional groups could also realize the pH responsiveness. Chitosan (CS) on the Fe_3_O_4_@OCMC@ IRMOF-3/FA (Chowdhuri et al., 2016), CS/Bio-MOF (Abazari et al., 2018), and hydroxyapatite (HAp) on the Fe_3_O_4_@Fe-MOF@Hap (Yang et al., 2017b) were applicable for drugs on-demand release at lower pH.

#### 2.3.2 Redox Responsiveness

Redox environment such as a high level of GSH, especially in the tumor, is preferable for controlled release through constructing redox-responsive carriers. MOFs, by assembly with disulfide bond-containing ligands, or dissolution through redox reaction upon redox agents, or easy modification with sensitive molecules, are such materials that meet the requirements. For example, *in vitro* released results showed that cargoes in MOF-M (DTBA) (M = Fe, Al, or Zr) consisted of metal ions and 4,4′-dithiobisbenzoic acid (4,4′-DTBA) displayed faster release in the condition of GSH, which could be attributed the disulfide bond in 4,4′-DTBA being cleavable (Lei et al., 2018). In addition, a new type of drug delivery system based on thiol-functionalized Uio-66-(SH)_2_ was developed for redox sensitiveness. In this system, benzoic acid (BA), as a modulator, competed with ligand 2,5-disulfanylterephthalic acid (BDC-(SH)_2_) to Zr6 clusters, thus forming small and large pores of Uio-66-(SH)_2_ after removal of BA. Then thiol-containing anticancer drug 6-mercaptopurine (6-MP) was loaded through a disulfide bond formed between thiol groups on BDC-(SH)_2_ and 6-MP. Upon high concentration of GSH, the release of 6-MP was shown (Gong et al., 2020). In another study, Wang et al. selected Cu(II) carboxylate-based MOF-199 to carry PSs. Once internalized by cells, Cu(II) reacted with and depleted endogenous GSH, induced decomposition of MOF-199, and released the PSs to ablate the tumor cells specifically (Wang et al., 2019d).

#### 2.3.3 Other Responsive Systems

Apart from widely used pH and redox conditions, other stimuli such as ATP, light, and temperature are also applied for controlled release. Adenosine triphosphate (ATP) is the energy source of all biological processes, whose dysfunction is related to many diseases, especially cancer. Therefore, developing ATP-responsive nanosystems depending on the interaction between ATP and counterpart is beneficial for intelligent drug release. ZIF-90, upon ATP, would be degraded due to competitive binding between ATP and Zn^2+^, thus releasing the cargo (Yang et al., 2019). Another strategy such as disrupting the coordination between ATP and anti-ATP aptamer is also available. In a DOX-loaded MOF coated with nucleic acid-based polyacrylamide hydrogel, anti-ATP aptamer on the MOFs could bind to ATP overexpressed in cancer sites and thus realize the controlled release of DOX (Chen et al., 2018b). Light-responsive MOFs synthesized by assembly metal Zr with photo-responsive linker azobenzenedicarboxylate (AZB) were stable in the dark, while they were rapidly degraded upon irradiation with light at 340 nm (Roth Stefaniak et al., 2018). Also, a plasmonic core–shell gold nanostars/ZIF-8 was light-responsive. Under near-IR (NIR) light irradiation, core gold nanostars created local temperature gradients and induced drug thermodiffusion (Carrillo-Carrion et al., 2019). Furthermore, redox-responsive selenium (Se)-substituted polymer coated onto porphyrin Zr-MOF (PCN-224) (Luo et al., 2019) also realized photo-induced release of the payload. Given the differential expression of enzymes at disease sites such as azoreductase, Fe-MOF composed of Fe^3+^ and 4,4′-azobisbenzoic acid was introduced for enzyme-responsive MOF. This MOF could be decomposed by reducing the azobenzene units to an amine by overexpressed azoreductase in an oxygen-deficient environment, thus releasing the payloads in cancer lesion sites (Liu et al., 2019a; Huang et al., 2019).

#### 2.3.4 Multi-Responsive Nanosystems

A single-responsive nanoplatform does not usually meet the precise release of payloads. Ren et al. prepared a pH/redox dual-responsive nanocarrier with an organosilica shell coated on ZIF-8. In an *in vitro* experiment, the release of DOX was only about 18% under neutral and DTT-free conditions, which increased to more than 38% upon 10 mM of DTT. When acidic and reduction conditions were simultaneously applied, more than 85% release of DOX was observed (Ren et al., 2019). For diabetic patients, excessive dosage of insulin would induce severe adverse effects, such as seizures and blindness. Therefore, intelligent carriers with the precise release of insulin according to glucose concentration are imperative. Chen et al. introduced such sense-and-treat carries, with ZIF-8 loaded with GOx and insulin. In the presence of glucose, GOx catalyzed glucose to produce gluconic acid, which triggered the disruption of ZIF-8 and then released insulin for treatment. Meanwhile, encapsulation with GOx and anti-vascular endothelial growth factor aptamer (VEGF aptamer) simultaneously in this system could be used for macular diseases (Chen et al., 2018c).

### 2.4 Other Functional Modification

Poly(ethylene glycol) (PEG), widely used in most nanomaterials, is applicable to extend drug release, colloidal stability, circulation time, etc. Coating PEG or its composites have been validated for excellent dispersibility, colloidal stability, and long half-lives in Uio-AZB (Roth Stefaniak et al., 2018), Hf-porphyrin MOF (dopamine-derived PEG) (Liu et al., 2018), and Uio-66 (Abanades Lazaro et al., 2017; He et al., 2019b). In addition, in an experiment to deliver drugs by iron-carboxylate MOF (MIL-101-Fe), PEG coating was also able to lengthen the drug release time significantly (Gupta et al., 2019).

Cyclodextrins (CDs) or their derivatives are water-soluble cyclic oligosaccharide with a unique structure of hydrophilic exterior and hydrophobic interior. Anchoring them onto the surface of MOF would endow MOF with the ability to interact with different moieties such as drugs, PEG, and target ligands, thus extending their functions such as controlled drug release and longer half-time. Core–shell NPs, CDs@MIL-100 (Fe), not only retained the virtues of MIL-100 but also extended the applications such as protecting DOX-loaded MIL-100 against aggregation and grafting fluorescent molecules to MIL-100(Fe) (Qiu et al., 2020).

PDA, apart from the application as a photoacoustic (PA) contrast agent, exhibits significant affinity towards interfaces and thus is increasingly considered as a surface functional moiety. PDA-modified MIL-100 was able to improve the dispersibility and stability of NPs except for photothermal conversional efficiency (Zhang et al., 2018e; Feng et al., 2019). Other functional moieties, such as dextran (Lai et al., 2019), lipid (Li et al., 2020b), and glucose (Zhang et al., 2019b), also endowed beneficial effects for MOFs.

## 3 Therapeutics

### 3.1 Cancer

Currently, MOFs are most abundantly used for cancer treatment of all MOF-related nanoformulations. Except for commonly used chemotherapy and radiotherapy, dynamic therapy, immunotherapy, gene therapy, starvation therapy, and combinations are also increasingly being adopted, in which MOFs exert their role as carriers, therapeutic agents, or both (Supplementary Table 1).

#### 3.1.1 Chemotherapy

Chemotherapy is still the most routine therapy for cancer, and drugs commonly applied include DOX, 5-fluorouracil (5-FU), *cis*-platinum, camptothecin (Cam), paclitaxel (PTX), dichloroacetate (DCA), and curcumin (CCM). However, all of these drugs have their limitations, such as poor solubility. MOFs or their composites with various superiorities have been successfully used to deliver these drugs. With high surface area and large porosity, MOFs including ZIF-8 (Tiwari et al., 2017; Dong et al., 2019), MIL-100 (Cabrera-Garcia et al., 2019), and Uio-66 (Gong et al., 2020) have been widely investigated for loading of the abovementioned drugs with high loading efficacy. Apart from carriers of bare MOF, various MOF composites were attempted for enhanced chemotherapy. Multifunctional catalytic micromotor ZIF-67/Fe_3_O_4_/DOX constructed by DOX- and Fe_3_O_4_-loaded ZIF-67 displayed up to 58% drug loading ratio. Furthermore, under hydrogen peroxide (H_2_O_2_)-based catalytic reaction and external magnetic field, precise delivery and controlled release of DOX could be realized (Wang et al., 2018a). ZIF-8-PAAS nanocomposites by employing PAAS (poly(acrylic acid sodium salt)) as soft templates were successfully used as size-controlled and surface tunable nano-drugs (Yan et al., 2017). Other composites by integrating MOFs with phosphorylcholine-based zwitterionic copolymer for longer circulation time, with carboxymethylcellulose (CMC) biopolymer for oral delivery, were exploited and used for enhanced tumor ablation (Javanbakht et al., 2020; Xie et al., 2020).

To overcome lower anticancer efficacy caused by multiple drug resistance, co-delivery of two or more drugs by MOFs was introduced. Zhang et al. first introduced a multi-drug carrier, PEG-FA/(DOX+VER)@ZIF-8, through encapsulating DOX and verapamil hydrochloride (VER) into ZIF-8 by one-pot process, followed by modification with PEG-FA. Results showed that drug loading content of DOX and VER was approximately 8.9% and 32%, and IC_50_ of PEG-FA/(DOX+VER)@ZIF-8 was much lower than that of free DOX and DOX@ZIF-8 in both MCF7/A and B16F10 cells. Upon modification with PEG-FA, this nanoparticle could be increasingly accumulated in tumors and enhanced the therapeutics (Zhang et al., 2017c). Similarly, ZIF-90 with DOX covalent to surface and 5-FU encapsulation into pores showed that drug loadings were as high as 36.35 and 11–13.5 wt% for 5-FU and DOX, respectively (Zhang et al., 2017a). In another cancer ablation experiment, quercetin (Que) and DOX co-loaded to HA/ZIF-8 were proved to synergistically enhance anticancer efficacy through remodeling tumor microenvironment by Que (Li et al., 2019).

#### 3.1.2 Immunotherapy

Immunotherapy, based on activating a specific self-immune system, is a burgeoning and promising therapy for cancer. Up to now, as antigen transportation platform for tumor-associated antigens (TAAs) and/or immune adjuvants, MOFs were increasingly used for tumor treatment, where TAAs were delivered to antigen-presenting cells (APCs) to induce CD8^+^ cytotoxic T lymphocyte responses, and adjuvants were used to amplify this response. As carriers, ZIF-8 exhibited high loading of cytosine-phosphate-guanine oligonucleotide (CpG), and the resultant ZIF-8/CpG complexes could induce more cytokine production and thus exerted potent immunostimulatory roles (Zhang et al., 2017b). Amine-functionalized zirconium-based MOF (Uio-AM) was proved to activate the complement systems (Qi et al., 2019), which could become a safe vaccine carrier. Further co-delivery of TAAs and adjuvants CpG ODNs by ZIF-8 (Zhang et al., 2017b) and MIL-101-Fe-NH_2_ (Yang et al., 2018b) demonstrated a stronger cellular immune response. Interestingly, another strategy of immunotherapy, by delivery of checkpoint inhibitors nivolumab (NV) by ZIF-8 followed by coating cancer cell membrane, was also demonstrated to be effective for both hematological malignancies and solid tumors (Alsaiari et al., 2021).

#### 3.1.3 Gene Therapy

Gene therapy, a personalized therapy based on regulating genes expressed specifically in diseases, has gained more and more attention. Until now, MOFs have been reported successfully for carrying small interfering RNA (siRNA), Cas9/sgRNA compounds, and plasmids. Zhuang et al. have reported a platelet cell membrane-coated ZIF-8 as a siRNA delivery platform, and high silencing efficiency was achieved *in vitro*. Excitingly, effective tumor targeting and therapeutic efficacy were also observed in *in vivo* experiments (Zhuang et al., 2020). Similarly, selenium/ruthenium nanoparticles modified MIL-101(Fe) loading pooled siRNAs (P-gp siRNA+VEGF siRNA) via surface coordination, which was demonstrated with enhanced cancer ablation (Chen et al., 2017b). Besides, MOF such as ZIF-8 and ZIF-90 have been fascinatingly applied in the delivery of the CRISPR/Cas9 genome editing system, which is a potential direction for disease treatment (Yang et al., 2019; Alyami et al., 2020).

#### 3.1.4 Dynamic Therapy

Dynamic therapy, killing cancer cells based on cytotoxic reactive oxygen species (ROS) produced by stimuli such as light, Fenton reaction, and ultrasound (US), includes PDT, chemodynamic therapy (CDT), and sonodynamic therapy (SDT).

##### 3.1.4.1 Photodynamic Therapy

PDT, a noninvasive therapeutic strategy based on light, has been widely used for cancer. After light energy is transferred to PSs, ROS are produced to kill cancer. With higher surface areas, MOFs could deliver large amounts of PSs. In addition, MOFs could be fabricated with PSs as organic linkers. Both strategies could make MOFs conductive in PDT. Until now, various PSs such as porphyrin derivatives (Hynek et al., 2018; Kan et al., 2018), Ce6 (Fu et al., 2020), BODIPY (Wang et al., 2016; Guan et al., 2018), TMPyP4 (Meng et al., 2018), and ZnPc (Xu et al., 2018) have been successfully loaded in different MOFs for PDT. MOFs constructed with porphyrin derivatives as organic linkers were also widely used for PDT (Ma et al., 2017; Zhou et al., 2018b; Gao et al., 2019). For example, the Ce6 delivery system based on ZIF-8 with modification with HA exhibited ultrahigh Ce6 encapsulation capability (76.8%). And this nanoparticle killed about 88.4% of cancer cells upon irradiation (Fu et al., 2020). Furthermore, without any drug loading, Sm-H_2_TCPP themselves synthesized by Sm^3+^ as metal nodes and PSs tetrakis (4-carboxyphenyl)porphyrin (H_2_TCPP), as organic linkers were demonstrated with prominent ROS generation capacity (Gao et al., 2019).

Considering that the hypoxic microenvironment contributes to the unsatisfactory effect of PDT, various strategies, including producing extra oxygen (O_2_), reducing the O_2_ consumption, and decreasing the GSH levels, were conducted to improve PDT efficiency. Core–shell nanosystem RC@TFC, with RAP-Ce6 composites as core and CAT-loaded Fe-MOF as shell, could improve hypoxia environment through catalyzing abundant H_2_O_2_ to O_2_ by CAT and inhibiting expression of hypoxia-inducible factor-1α by rapamycin simultaneously, thus potentiating the PDT (Liu et al., 2019b). Other O_2_-evolving approaches such as MOF-nanozyme composites, including Au@ZIF-8 (Ma et al., 2019) and PCN-224-Pt (Zhang et al., 2018d) nanostructures, were also able to overcome the hypoxic conditions and improve the PDT efficiency. In contrast, to improve the O_2_ supply, Chen et al. introduced another ZIF/PC complex, in which mitochondrial complex I inhibitor papaverine (PPV) was encapsulated and delivered to the tumor to reduce the intratumor oxygen consumption (Chen et al., 2021). As high-level GSH would also reduce the PDT effect, MOF-2 was designed with Cu(II) as an active center to absorb and lower GSH and then enhanced PDT (Zhang et al., 2018c).

Apart from the hypoxic environment, the penetration depth is another obstacle that limits the application of PDT. Shi et al. developed a nanosystem by decorating titanium dioxide nanoparticles on upconversion nanoparticle–MOF composites. Upon irradiation by 980-nm NIR light, upconversion nanoparticles could generate ultraviolet and visible lights and induce titanium dioxide and porphyrin to produce ROS through Type I and II PDT. More importantly, this nanosystem could realize deep tissue penetration (Shi et al., 2020).

##### 3.1.4.2 Chemodynamic Therapy

CDT, an emerging therapeutic approach based on tumor environment, is a kind of treatment based on *in situ* production of hydroxyl radical (·OH) via Fenton reaction and Fenton-like reaction. In CDT, transition metal ions such as Fe^2+^, Cu^2+^, and Mn^2+^ catalyze H_2_O_2_ to ·OH and kill tumors. Therefore, MOFs constructed by these metal ions could be used for CDT. Moreover, increased H_2_O_2_ levels and decreased GSH levels are also beneficial for CDT. Wu et al. first introduced an autocatalytic nanoreactor through coating CuMOF on GOx-loaded dendritic mesoporous organosilica nanoparticles (DMONs). Once entering cells, CuMOF was disrupted in acidic endo/lysosomes. On the one hand, GOx catalyzed glucose to generate H_2_O_2_; on the other hand, Cu^2+^ induced GSH depletion and reduced Cu^2+^ to Cu^+^, both of which were important for self-boosting CDT (Wu et al., 2020). Other approaches without transition metal were also acceptable for CDT. By embedding horseradish peroxidase (HRP) and GOx into ZIF-8, a cascade catalytic bioreactor was reported against the solid tumor. After degradation of ZIF-8 in acidic conditions of cancer, HRP and GOx were released. Released GOx catalyzed glucose to produce H_2_O_2_, which was further catalyzed by HRP to ·OH (Bai et al., 2019). Nanozyme-MOF complex, for example, PEG-modified Cu-Pd@MIL-101(CPMP) with high POD-like and SOD-like activities, as well as the ability of depletion of GSH could produce a high level of hydroxyl radicals and thus be effective for tumor treatment (Yang et al., 2021b).

##### 3.1.4.3 Sonodynamic Therapy

SDT is a burgeoning and promising noninvasive therapy due to its superiority of deeper penetration. Under low-intensity US and sonosensitizers, ROS are produced to kill cancer. As in PDT, by loading sonosensitizers such as Ce6 in MOF, SDT for cancer ablation could be achieved under US (An et al., 2020a). In addition, MOF based on active components harboring the capacity of sonosensitizers including porphyrin derivatives (Xu et al., 2021) and Ti (Liang et al., 2021) could also be used for SDT.

#### 3.1.5 Others

Photothermal therapy (PTT), another noninvasive phototherapy, exerts its function through converting light energy to thermal energy, in which photothermal agents (PTAs) play an essential role during the progress (Hussein et al., 2018). The role of MOFs in PTT is mainly as carriers of PTA or as PTA.

As a widely used carrier, ZIF-8 has been also reported for successful loading autophagy inhibitor for tumor treatment through the autophagy pathway (Chen et al., 2018e). Both *in vitro* and *in vivo* experiments showed that 3-methyladenine (3-MA)-loaded MOFs could effectively inhibit the tumor as compared with free 3-MA.

Ferroptotic therapy, a novel mechanism of cell death through regulating different pathways such as lipid peroxide, has also attracted increasing attention in antitumor. As this therapeutic modality is dependent on iron ions, MOFs based on Fe or Fe-including could enhance the sensitivity of ferroptosis. By combining Fe-MOF with ferroptosis inducer, the efficacy of the antitumor would be enhanced (Liu et al., 2021b; Xin et al., 2021).

#### 3.1.6 Combination Therapy

**FIGURE 5:**
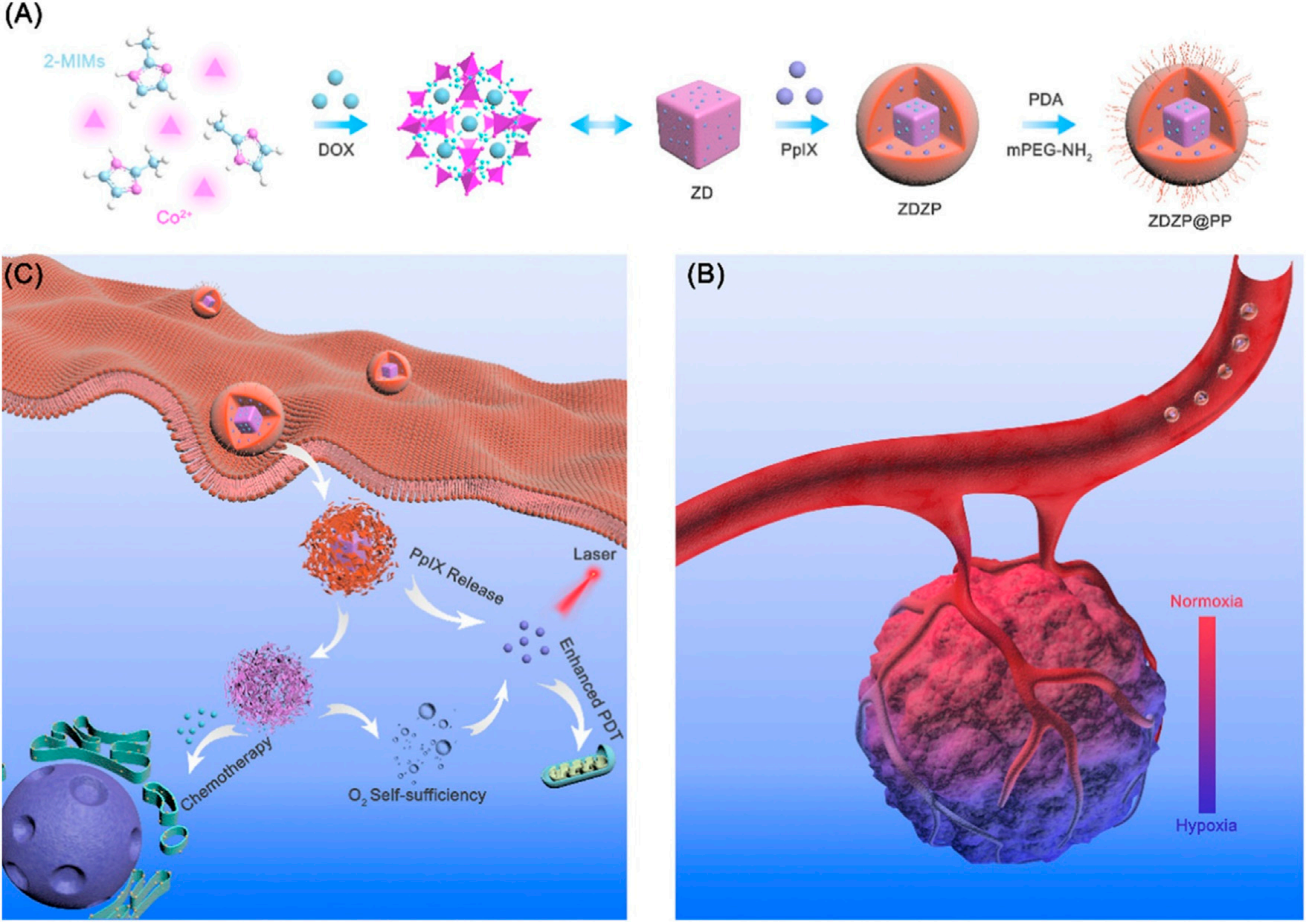
Schematic of ZDZP@PP NPs in photodynamic therapy (PDT)/chemotherapy combination therapy for tumor. **(A)** Synthesis of ZDZP@PP Nanoparticles. **(B,C)** Schematic illustration of ZDZP@PP in combination therapy. Reproduced with permission. Copyright 2020, *ACS Applied Materials & Interfaces*.

Compared with monotherapy, which often fails to achieve a desirable therapeutic effect, combination therapies may be more effective. Currently, various combination therapies such as chemotherapy/PDT (Liu et al., 2018; Ren et al., 2020), PDT/PTT (Gao et al., 2018a; Wang et al., 2019c), PDT/CDT (Wu et al., 2021a), and PDT/immunotherapy (Lan et al., 2018; Cai et al., 2020; Ni et al., 2020) have been increasingly applied. In this way, MOFs deliver different functional agents to kill tumors in different ways. pH-responsive nanoprobes, AuNCs@MOF-DOX, were constructed by loading PS gold nanoclusters (AuNCs) and DOX into ZIF-8 for simultaneous chemotherapy/PDT. In the tumor acidic conditions, DOX and AuNCs were released. Under irradiation, a large amount of singlet oxygen was produced and further enhanced the chemotherapy. Excitingly, compared with a single therapy, this combination completely eliminated the tumor in an *in vivo* experiment (Zhang et al., 2020b). In another multifunctional core–shell nano-agent (ZDZP@PP), PS protoporphyrin IX (PpIX)-loaded ZIF-8 was grown on the ZIF-67/DOX followed by PDA-PEG coating. Once internalized by cancer cells, PDA and ZIF-8 were disrupted with PpIX and ZIF-67 released. ZIF-67 as an H_2_O_2_ catalyst catalyzed H_2_O_2_ to oxygen and improved the PDT. Meanwhile, DOX from ZIF-67 killed cancer cells and thus realized oxygen self-sufficient chemotherapy/PDT combination (Ren et al., 2020) (Figure 5). Compared with MOFs only used as carriers, active moiety-containing MOFs such as PSs-MOF could potentiate the effect of treatment. For instance, hypoxia-activated prodrug (tirapazamine (TPZ))-loaded Hf-porphyrin MOFs have high porphyrin loading and produced higher ROS for PDT, which depleted oxygen and induced activation of TPZ for enhanced PDT/chemotherapy (Liu et al., 2018).

In addition, PDT/PTT is also the most common therapy. By gas absorption and modifiable properties, a biomimetic O_2_-evolving nanoplatform was fabricated. In this system, O_2_-loaded Uio-66 was conjugated with ICG as both PSs and PTAs, followed by RBC membrane coating. Upon irradiation, the photothermal property of ICG facilitated O_2_ release and then enhanced the PDT/PTT (Gao et al., 2018a). In another exciting experiment for tumor ablation, nano-agents (Zn-TCPP) constructed by Zn^2+^ as metal nodes and TCPP as organic linkers were used as PTAs more often than PSs, which benefited from large π electron conjugated in aromatic macrocycle (Wang et al., 2019c).

### 3.2 Inflammatory Diseases

In most recent years, researchers have seen the germination and development of MOFs in inflammation-related diseases. In summary, the main applications of MOFs in inflammation or inflammatory conditions are as follows: 1) the inherent therapeutics of MOFs, 2) MOFs as delivery systems or active carriers of drugs and antioxidant enzymes, and 3) nanozymes based on MOFs (Supplementary Table S2).

MOFs exert their role in inflammatory disease mainly as drug carriers. Supramolecular γ-CD-MOFs as carriers of sodium diclofenac (DFNa) showed approximately 50% entrapment efficiency and more prolonged controlled release of drugs. In carrageenan-induced mouse paw edema, the inhibition rate was 88.7% after treatment with γ-FeCD-MOF (Abucafy et al., 2018). As for microenvironment and pathogenesis, Xiong et al. engineered an intelligent pH-responsive drug delivery system modified by hydrophilic agent HA, which could improve the hydrophilicity of MOFs and inflammation of OA simultaneously. In the weak acidic environment of osteoarthritis (OA), MIL-100(Fe) was disrupted, and protocatechuic acid (PCA) was released for alleviating inflammation by reducing the inflammatory mediators (Xiong et al., 2020). Similarly, as mentioned above, an oxidation-responsive MOF, Ce-MOF@PSS, was designed based on the microenvironment of inflammatory bowel disease (IBD) and antioxidant ability of Ce^3+^/Ce^4+^. By utilizing the negatively charged properties of poly(sodium-4-styrenesulfonate) (PSS), the obtained Ce-MOF@PSS adhered to the inflammatory intestine. In inflammatory sites, the reaction between ROS and Ce^4+^ eliminated ROS and induced the pore size transformation of Ce-MOF, thus releasing drugs. Apart from the excellent performance of inflammation-targeting ability in different colitis models, significant alleviation of inflammation was observed in spontaneous colitis (Yin et al., 2021).

**FIGURE 6:**
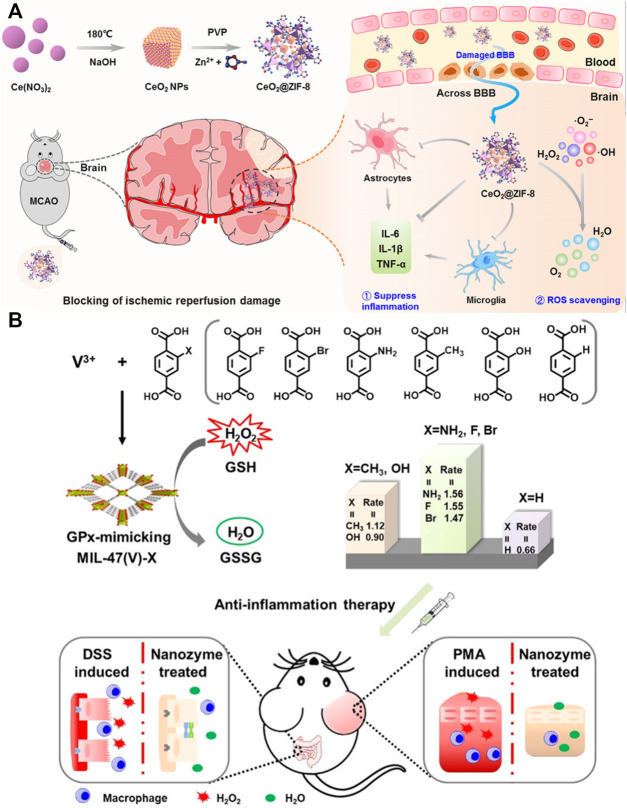
Schematic of nanozyme based on metal–organic frameworks (MOFs) for inflammatory diseases treatment. **(A)** Schematic of CeO_2_@ZIF-8 synthesis and application mechanism against ischemic stroke. Reproduced with permission. Copyright 2020, *Science Advances*. **(B)** Schematic of designing principle and application in the anti-inflammatory treatment of GPx-mimicking MIL-47(V)-X-MOF nanozymes. Reproduced with permission. Copyright 2021, *Angewandte Chemie* International Edition.

Antioxidant enzymes, with the capacity of catalyzing cytotoxic free radicals to nontoxic ingredients, are also effective in inflammatory diseases. Using natural enzymes including superoxide dismutase (SOD) and CAT into PCN-333(Al) was proved to protect cells from toxic ROS for up to a week as compared with free enzymes with only a short duration, which have the potential for treating inflammation (Lian et al., 2017). Similarly, He et al. constructed bioactive ZIF-8-capped ceria NPs (CeO_2_@ZIF-8 NPs) for reperfusion-induced injury in ischemic stroke. As expected, these NPs effectively prevented and improved ischemic stroke by inhibiting lipid peroxidation and reducing oxidative damage and neuron apoptosis (He et al., 2020) (Figure 6A). Wu et al. further introduced a glutathione peroxidase (GPx) mimicking MIL-47 (V)-NH_2_ nanozyme based on MIL-47 (V) through ligand engineering strategy. Both *in vitro* and *in vivo* experiments demonstrated excellent antioxidant capacity, which provided a new direction for inflammation treatment (Wu et al., 2021b) (Figure 6B). More interestingly, Mg/COOH-MOF without carrying any other functional agents showed excellent anti-inflammatory activity and is beneficial for OA treatment (Li et al., 2020c).

In a recent study for managing severe sepsis, Liu et al. designed a cationic MOF by grafting cationic polyethylenimine (PEI) to ZIF-8. As a “nanotrap,” this MOF could scavenge circulation cell-free DNA (cfDNA) produced by damaged cells and inhibit cfDNA-induced TLR activation and other signaling pathways, thus alleviating the inflammation in sepsis, which provided a novel strategy for inflammation treatment (Liu et al., 2021a).

### 3.3 Antibacteria

Bacterial infection has become a public burden, which causes increased mortality and morbidity worldwide, with inadequate treatment induced by antibiotics abuse. Hence, it is crucial to seek newer and more effective ways for bacterial eradication.

Drugs, especially antibiotics, are still the main therapeutics. However, the hydrophobic nature, frequent demonstration, and the resultant drug resistance reduce the effect of drugs. MOFs as carriers of antibiotics have solved these problems. Until now, vancomycin (Van) (Chowdhuri et al., 2017), ceftazidime (Sava Gallis et al., 2019), ciprofloxacin (CIP) (Esfahanian et al., 2019), tetracycline (Tet) (Zhang et al., 2019d), and rifampicin (Ahmed et al., 2020) have been successfully encapsulated into ZIF-8 and its composites for antibacterials with intelligent drug release. For example, ceftazidime@ZIF-8 particles by encapsulating antibiotics into ZIF-8 showed sustained release of ceftazidime for up to a week, and 50 μg/ml of nanoparticles inhibited Gram-negative *Escherichia coli* completely (Sava Gallis et al., 2019). Interestingly, in another delivery system, zinc ions released from ZIF-8 under an acidic environment exhibited a synergistic antibacterial effect (Zhang et al., 2019d). Compared with loading antibiotics, Mao et al. designed a precise antibacterial system by perfusing metabolic labeling molecule 3-azido-*d*-alanine (*d*-AzAla) into MIL-100(Fe). Under H_2_O_2_ condition, *d*-AzAla was released and selectively adhered to the cell walls of bacteria, which recognized and reacted with additional dibenzocyclooctyne (DBCO)-modified PS NPs, thus achieving precise bacteria killing by selective FL labeling (Mao et al., 2018a).

**FIGURE 7:**
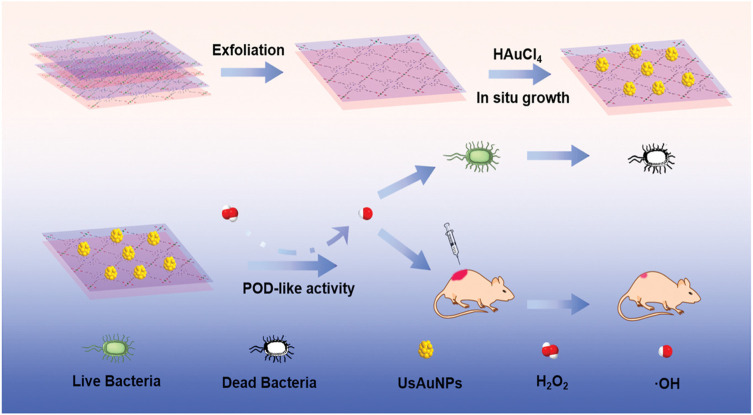
Schematic of UsAuNP/metal–organic framework (MOF) hybrid with POD-like activity for antibacterial therapy. Reproduced with permission. Copyright 2020, *Small*.

**FIGURE 8:**
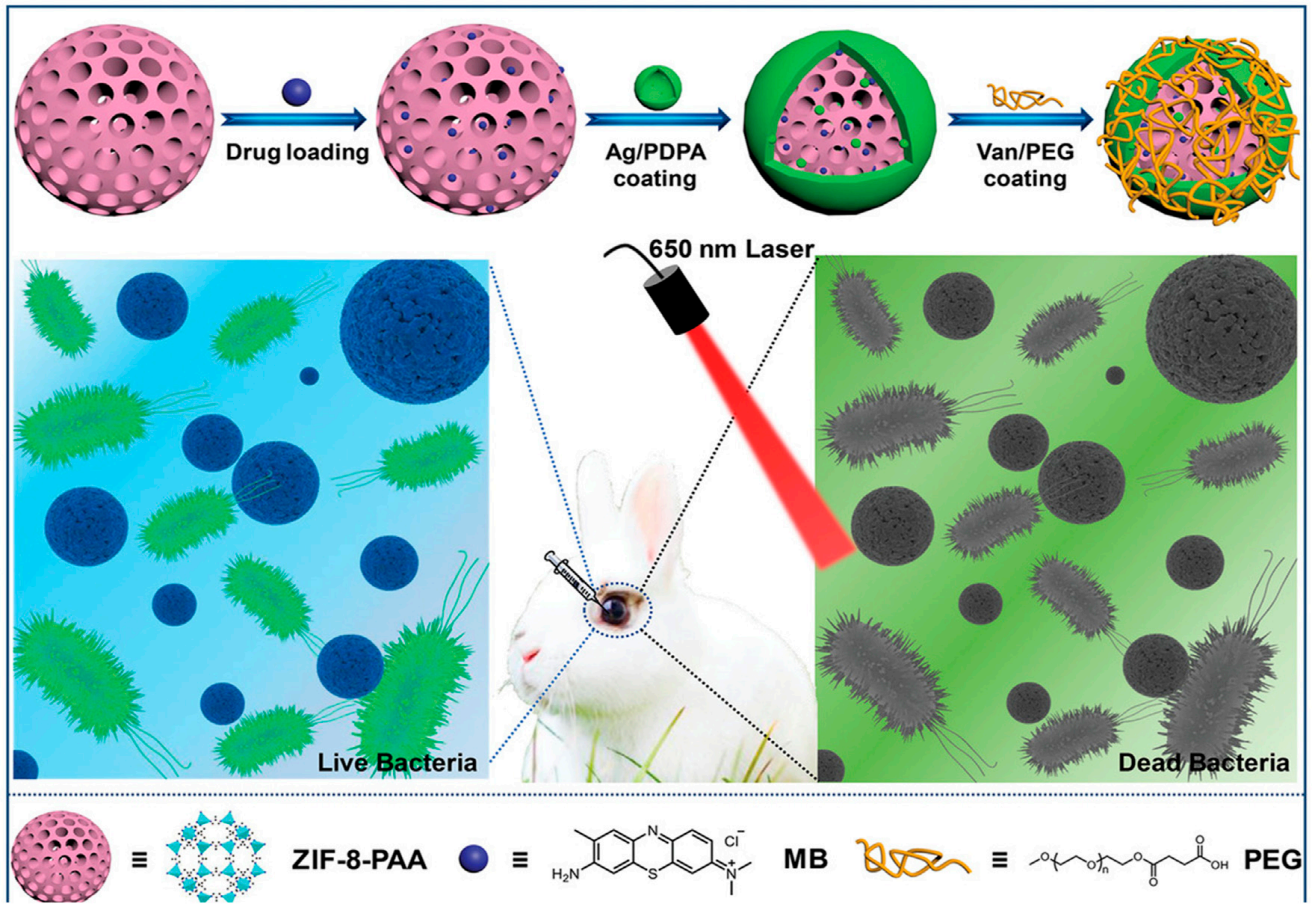
Schematic of ZPMAVP system for application in synergistic chemotherapy and photodynamic therapy for endophthalmitis. Reproduced with permission. Copyright 2019, *Small*.

The application of silver nanoparticles (AgNPs), another promising antibacterial approach, is hampered by their aggregation. Immobilization of AgNPs into γ-cyclodextrin MOFs (CD-MOFs) (Shakya et al., 2019; Luo et al., 2020) and Ag-doped magnetic MOF (γ-Fe_2_O_3_@SiO_2_@ZIF-8-Ag) (Rahmati et al., 2020) showed superior performance in bacterial inhibition. In addition, nanozyme-based AuNP/MOF hybrid with excellent POD-like activity exhibited significant antibacterial effects (Hu et al., 2020) (Figure 7). Li et al. constructed self-activated cascade MOF/enzyme composites with peroxidase-like activity based on GOx-loaded MIL-MOF, where GOx could reduce the pH value for optimum activity of MIL to inhibit the methicillin-resistant *Staphylococcus aureus* (MRSA) (Li et al., 2020a). In combination therapy, for example, ZIF-8@PDA composites as carriers of Van have been demonstrated to have effective bacterial eradication through NIR/pH-responsive synergistic PTT/chemotherapy (Xiao et al., 2021). Another composite nanomaterial, ZIF-8-PAA-MB@AgNPs@Van-PEG, exhibited an ability against three kinds of bacteria based on PDT/AgNP/chemotherapy (Chen et al., 2019a) (Figure 8).

In addition, as surface modification agents, MOFs incorporated into matrix materials such as CS are used to enhance the antibacterial properties. For example, Uio-66-NH_2_/CS composite by fusing UiO-66-NH_2_ with CS has peroxidase and oxidase mimicking activity and exhibited good antibacterial properties in both H_2_O_2_-present and H_2_O_2_-absent conditions. In the presence of H_2_O_2_, this composite killed bacteria through peroxidase-mimicking activity; however, without H_2_O_2_, it could also degerm under 30 min of UV pre-irradiation (Wang et al., 2021).

## 4 Imaging

Early diagnosis and monitoring of diseases by imaging technology are indispensable for timely precaution and treatment. Current imaging technologies include MRI, CT, optical imaging, PA imaging (PAI), and positron emission tomography (PET). MOFs as contrast agents or imaging agent carriers have been widely applied in imaging for tracing diagnosis or therapeutics (Supplementary Table S3).

### 4.1 Magnetic Resonance Imaging

MRI is a noninvasive image modality, which is based on the interaction between water proton nuclei of samples and external magnetic field (Foltz and Jaffray, 2012), in which contrast agents amplify the signal by enhancing the relaxivities of water protons. Magnetic metal ions such as Fe, Mn, and Gd are widely used as contrast agents. Among them, Gd and Mn are used as T_1_-weighted-enhanced agents, while Fe was used as T_2_-weighted MRI agents (Xiao et al., 2016). However, disadvantages such as low retention time and toxicity induced by frequent dosing of single metal significantly impede their application. With ideal size, high loading efficiency, and ease of modification, MOFs constructed with the above metal as nodes or with the above metal oxides loaded or as coating could be used for increasing retention time and relaxivities. For example, Fe_3_O_4_@UiO-66 composites, fabricated by growing UiO-66 on Fe_3_O_4_, exhibited good capability as contrast agents with transverse relaxivity (r2) of 1,396 mg mL^−1^ s^−1^, which was much higher than that of commercial Fe-based agents such as Resovist (150 nM^−1^ s^−1^) (Zhao et al., 2016). Other Fe-MOFs such as MIL family (MIL-53, MIL-100, etc.), Mn-MOF such as Mn-porphyrin MOF (PCN-222) (Zhang et al., 2018a; He et al., 2019a), MnO_2_-coated porphyrin-MOF (PCN224) (Zhang et al., 2019a; Min et al., 2019; Tian et al., 2019), and MOFs containing Mn^2+^ (Meng et al., 2021) also showed beneficial performance for MRI.

### 4.2 Computed Tomography

CT image is a common technology of diagnosis and treatment by visualizing the internal structures of the body, which is based on the X-ray attenuation after tissue absorption. The addition of contrast agents would enhance the contrast between lesion tissue and normal tissues. Apart from barium sulfate suspensions only applied in CT for the gastrointestinal (GI) tract, iodinated aromatic molecules are routine contrast agents for CT images. Still, they also cause short blood circulation and poor sensitivity (Lee et al., 2013). CT contrast agent Uio-PDT, formed by diiodo-substituted monocarboxyl-functionalized BODIPY (I_2_-BDP) with metal, not only overcame the limitation of iodinated molecules but also exhibited the best imaging performance after 24 h (Zhang et al., 2017d). Other high-Z elements, including Zr- and Hf-based MOFs, have also been evaluated as contrast agents for CT (Dekrafft et al., 2012).

### 4.3 Positron Emission Tomography

PET is an imaging technology for monitoring metabolism processes by tracing signals from radioisotopes (^89^Zr, ^64^Cu, ^68^Ga, etc.) doped into contrast agents (Derlin et al., 2018). Therefore, MOFs based on these metals with targeting modification would be beneficial for PET imaging. By incorporating positron-emitting isotope ^89^Zr into Uio-66 MOF, combining with PEG modification, and targeting peptide ligand (F3), Chen et al. examined the distribution and clearance profile of MOF *in vivo*. Both *in vivo* and *ex vivo* PET imaging results showed that tumor uptake of ^89^Zr-UiO-66 was faster and higher than in all other major organs, which exhibited potential tumor contrast (Chen et al., 2017a).

### 4.4 Optical Imaging

**FIGURE 9:**
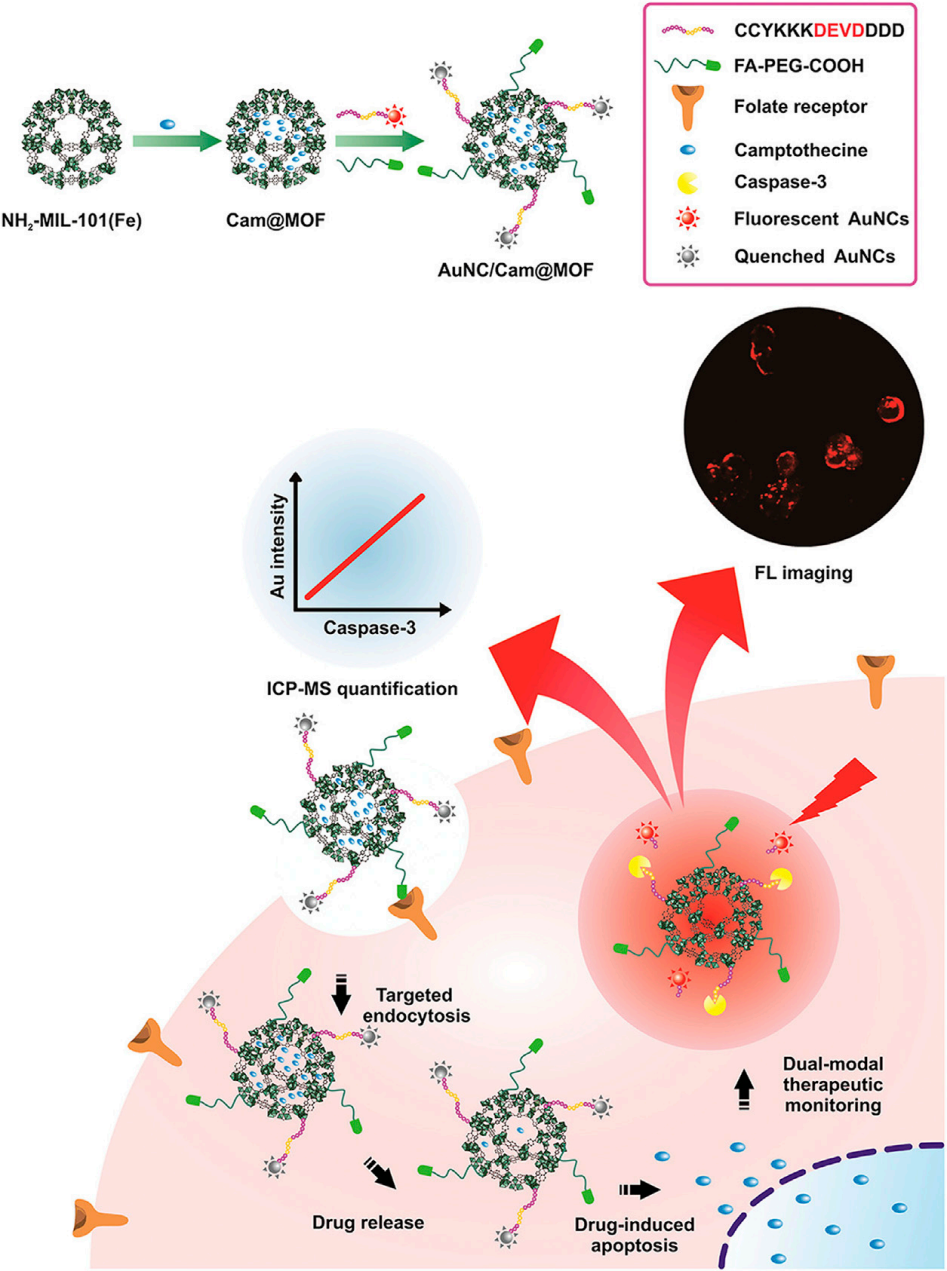
Diagram of construction of multifunctional AuNC/Cam@MOF nanoprobe and its applications including fluorescence (FL) imaging. Reproduced with permission. Copyright 2019, *Analytical Chemistry*.

Optical imaging provides opportunities for real-time monitoring of tissues at the microscopic level without tissue damage, including FL, phosphorescence, and chemiluminescence (CL) imaging (Pirovano et al., 2020). In optical imaging, FL is the most widely used. MOFs constructed with luminophore as metal nodes or organic linkers, loaded with FL dyes, and combined with other functional moieties including quantum dots (Qin et al., 2020), AuNPs (Yin et al., 2019), ruthenium(II)tris(bipyridyl) cationic complex (Ru (bpy)_3_^2+^) (Chen et al., 2017c) have been successfully applied. For example, Cy@ZIF-8, by incorporating NIR dye cyanine (Cy) to ZIF-8, not only improved the water solubility of Cy but also exhibited strong FL both *in vitro* and *in vivo* (Li et al., 2018). Yin et al. constructed a nanoplatform for real-time monitoring and quantifying the therapeutic drug response. In this system, fluorescent AuNCs were decorated onto Cam@MIL-101 (Fe) through a peptide, which could be cleaved by intermediate products of camptothecin. Once internalized by HepG2 cells, Cam induced the production of caspase-3, thus releasing AuNCs and restoring their FL. Meanwhile, released Au could be used to quantify the amount of caspase-3 (Yin et al., 2019) (Figure 9).

### 4.5 Photoacoustic Imaging

PAI has the superiorities of superb contrast, super spatial resolution, and deeper penetration based on the optoacoustic effect and contrast agent (Fu et al., 2019). Gold nanoparticles, dyes with NIR absorption including indocyanine green (ICG), and porphyrin derivatives were widely used for PA. The combination of these dyes with MOFs may enhance their performance. By integrating Au nanostar with ZIF-8, Deng et al. successfully fabricated a novel light-responsive yolk–shell nanosystem. Upon 1,064 nm (NIR-II bio-window) of laser irradiation, this nanosystem showed excellent photothermal conversion capability for PTT. In addition, with intense NIR absorbance, the potential of PAI was performed *in vivo*. Compared with weak signals from control, tumor sites of mice administrated with Au@ZIF-8 exhibited strong PAI signals at 12 h (Deng et al., 2019). In another experiment, ICG-engineering MIL-100(Fe) by integrating MIL-100 with ICG showed strong NIR absorbance. In addition, the solubility and targeting ability of ICG were improved. As expected, both *in vitro* and *in vivo* experiments demonstrated satisfactory results in PAI (Cai et al., 2017).

### 4.6 Multi-Modal Imaging

**FIGURE 10:**
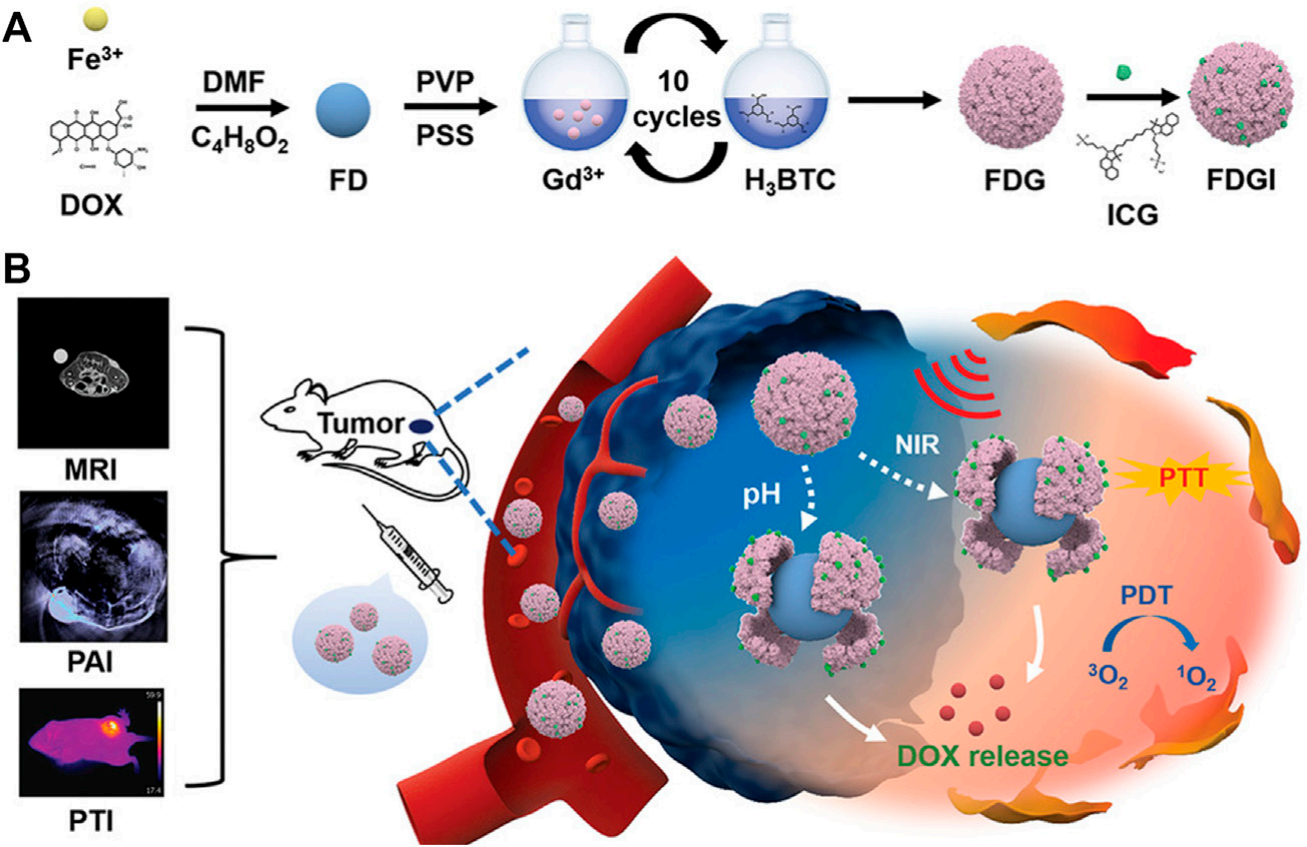
The synthesis and application of Fe-DOX@Gd-MOF-ICG nanoplatform (FDGI NPs) in MR/photoacoustic/photothermal (MR/PA/PT) multimodal imaging-guided therapeutics. **(A)** The synthesis scheme of FDGI NPs. **(B)** The application of FDGI NPs in MR/PA/PT imaging-guided therapeutics. Reproduced with permission. Copyright 2020, *Advanced Healthcare Materials*.

As none of these image modalities are fully qualified for disease monitoring due to the limitation of each modality, combining them with two or more technologies would be a better choice. For instance, in bioactive Fe-DOX@Gd-MOF-ICG nanoplatform, the Gd-MOF shell was used for MRI apart from the protective shell for Fe-DOX cores. Besides, both PAI and photothermal imaging (PTI) were successfully introduced by loading ICG, thus realizing the multimodal imaging-guided therapeutics (Zhu et al., 2020) (Figure 10). Other MOFs without loading dyes and modification could also achieve multi-modal imaging. For example, MOFs synthesized by integrating Ga^3+^ with fluorescent linker pDBI (pDBI = 1,4-bis(5-carboxy-1*H*-benzimidazole-2yl)benzene) without any other modification could be used for FLI and MRI simultaneously (Kundu et al., 2016).

## 5 Theranostics

Disease theranostics, a combination of therapeutics and diagnostics, has attracted increasing attention in personalized and accurate treatment for diseases, which simultaneously require the diagnosis and monitoring for the treatment processes.

Functional MOFs constructed by metals such as Hf, Fe, Cu, and Gd or/and organic linkers such as porphyrins could deliver therapeutic agents selectively to lesion sites with surface modification and thus quickly realize imaging-guided treatment. For example, the Hf-porphyrin platform with hypoxia-activated prodrug (TPZ) trapped and modified with dopamine-derived polymer (DOPA-PIMA-mPEG) was successfully used in CT imaging-assisted PDT/chemotherapy. In this system, by incorporating porphyrin into a platform, the efficiency of PDT was improved. Then, TPZ was activated under hypoxic conditions induced by PDT and further enhanced the treatment. Furthermore, after being intratumorally injected, an unmistakable enhanced CT signal was observed at tumor sites. Another “all-in-one” nano-agent based on Hf as metal cluster and Mn (III)-porphyrin as linkers with FA coating exhibited excellent performance both in treatment and in imaging. On the one hand, FA guided nano-agent to tumor sites, and then the CAT-like activity of Mn-porphyrin improved the hypoxic condition by catalyzing H_2_O_2_ to O_2_, which boosted the PTT/RT combination therapy. On the other hand, this system has high performance for MRI/CT/PAI enhancements. Signals from CT and MR were increased by 1.7-fold and 3- to 5-fold, respectively. Thus, this triple-modality imaging-assisted PTT/RT system showed high potential in tumor theranostics (Bao et al., 2020).

Similarly, MOFs as carriers could also realize multifunctional theranostics. Guo et al. engineered a pH/NIR dual-responsive nanocarrier based on ZIF-8 for MRI/PAI-aided chemo-phototherapy. In this system, DOX was embedded into ZIF-8 through a one-pot way, followed by modification with PDA, Mn ions, and PEG. Under NIR laser irradiation, the resultant ZIF-8/DMPP showed a satisfactory thermal effect, which accelerated the release of DOX and augmented the chemotherapy. Meanwhile, the chelation of Mn ions onto PDA shells made this system possible for MRI/PAI, which was proved by both *in vitro* and *in vivo* results (Guo et al., 2020).

## 6 Sensors

Monitoring abnormal changes of molecules during the pathologic process of diseases, such as biomacromolecules, as disease markers, is of great importance for understanding the severity and pathogenesis of diseases. In this chapter, we emphasized the applications of MOFs in biosensors from the following aspects: FL, CL, and colorimetric methods.

### 6.1 Fluorescence Detection

**FIGURE 11:**
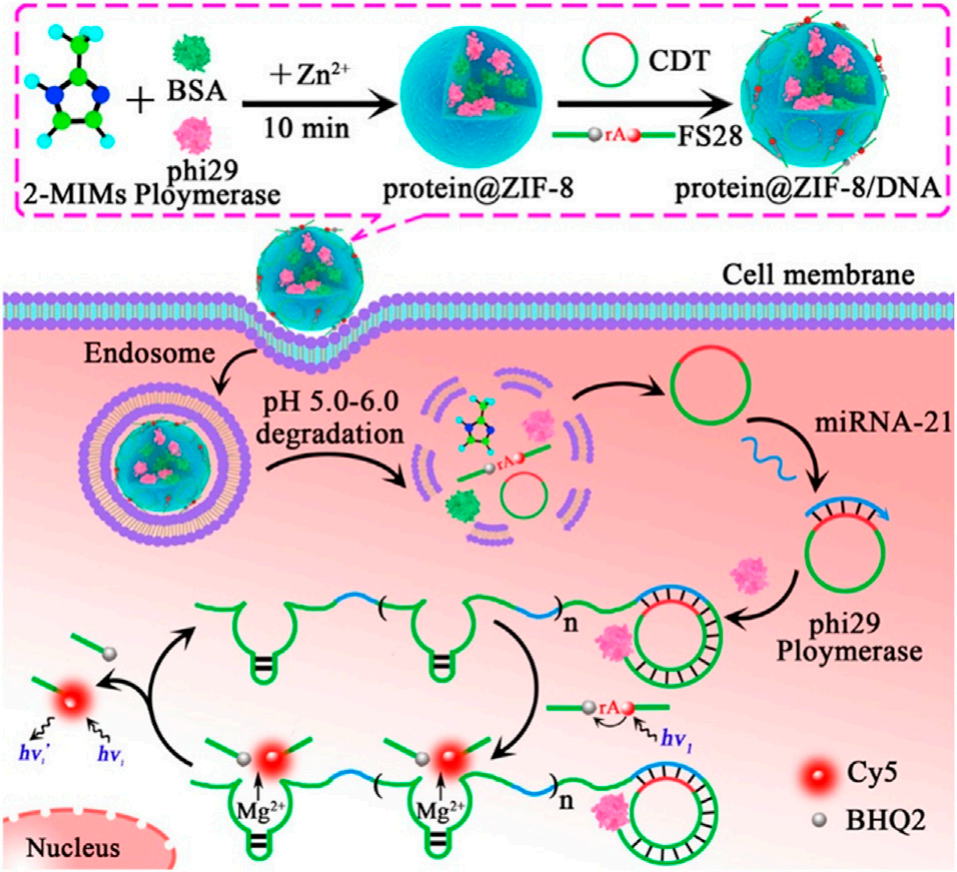
Illustration of fluorescence imaging for microRNA-21 by using ZIF-8 NPs as a nanocarrier for codelivery of ϕ29 DNA polymerase and DNA probes in living cells. Reproduced with permission. Copyright 2019, *Analytical Chemistry*.

As carriers, MOFs could deliver FL dye-labeled molecules such as DNA or RNAs to the reaction system. After cargoes in MOFs were reacted with other substances, FL signals were detected. For example, as carriers, ZIF-8 delivered ϕ29 DNA polymerase (ϕ29DP) and nucleic acid probes to cells, in which ϕ29DP was encapsulated into ZIF-8, and nucleic acids were absorbed onto ZIF-8. ϕ29DP and nucleic acid probes were released in acidic cellular conditions. In the presence of ϕ29DP and deoxyribonucleotide triphosphates (dNTPs), the intracellular miRNA-21 triggered a rolling circle amplification (RCA) reaction and produced an autonomous resultant Mg^2+^-dependent DNAzyme, which cleaved the fluorogenic substrate and provided a readout FL signal for the monitoring of miRNA-21 (Zhang et al., 2019c) (Figure 11).

**FIGURE 12:**
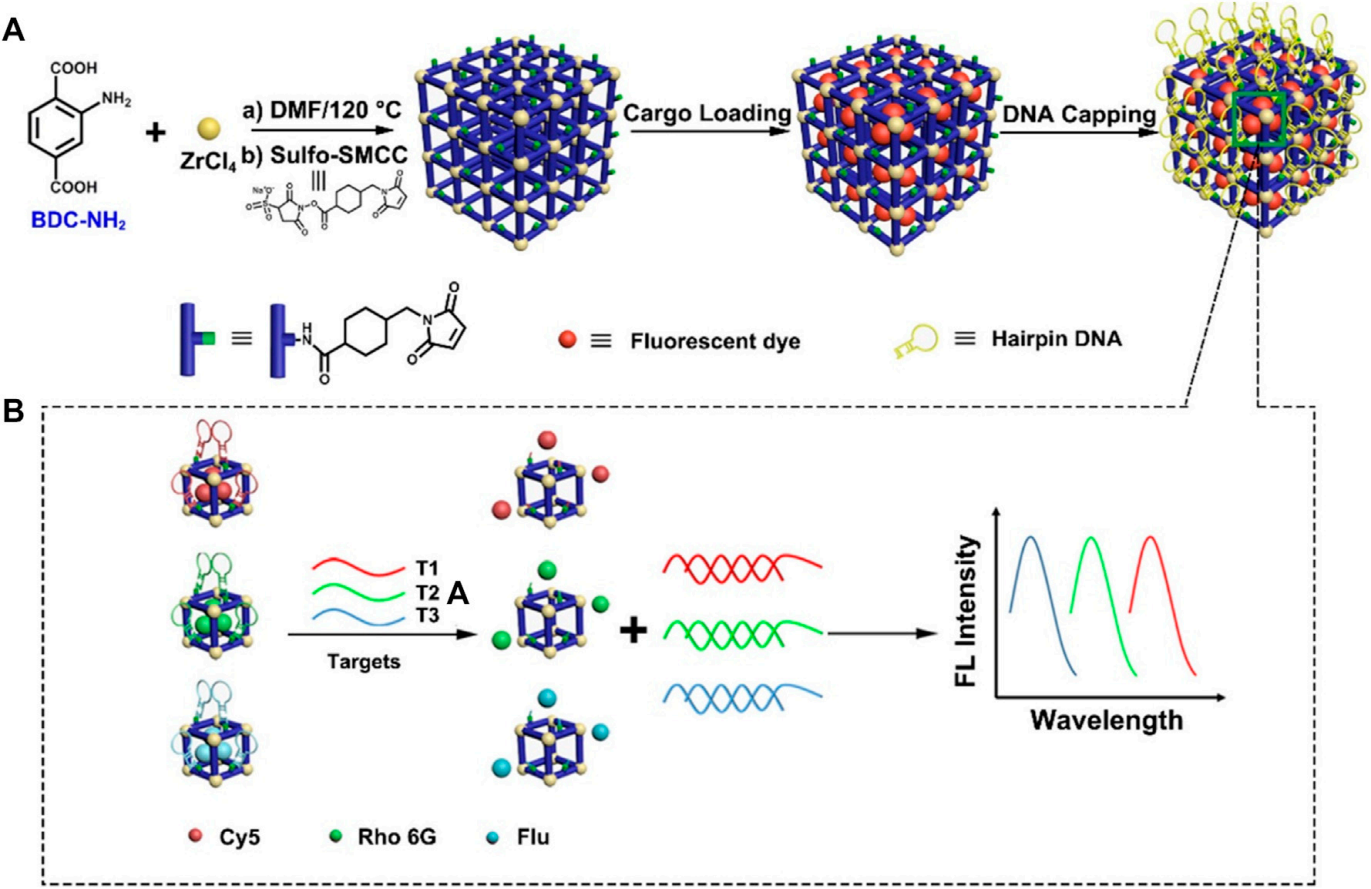
The synthesis and application of metal–organic framework (MOF)-based nanoprobes in DNA multicolor detection. **(A)** Schematic diagram of synthesis of MOF-based nanoprobes. **(B)** Diagram of MOF-based probes in multicolor detection of DNA targets. Reproduced with permission. Copyright 2018, *Analytical* *Chemistry*.

Except for carriers, MOFs could also be designed as a “gate-keeper” for switch-on/off of fluorophore in FL detection. Wu et al. designed a multicolor detection system with signal dyes loaded and DNA hairpin structure as a capping shell. In this system, as 3′-end of single-strand hairpin-DNA was complementary to DNA probes, FL signals of DNA probes were switched off by the stem-loop structure of hairpin DNA. However, once DNA targets were introduced, FL dyes would be released through a competitive reaction. DNA targets could be detected by monitoring the FL signals with a low detection limit of 20 fM. Meanwhile, by loading three different FL dyes with corresponding hairpin DNA, this system enabled multiple DNA detection simultaneously (Wu et al., 2018) (Figure 12). This “switch-on” FL detection is also applicable for other biomolecules. In the platform of AgNPs@PCN-224, AgNPs as a “quencher” reduced the FL of PCN-224; once contacted with H_2_O_2_, AgNPs were etched into silver ions and restored the FL of PCN-224 (Du et al., 2020). Similarly, “switch-off” FL detection achieved by fluorescent MOF such as IRMOF-3 without any modification was also feasible. Azoreductase-responsive AMOF@MBs with TAMRA (carboxytetramethylrhodamine, donor) and Cy5 (acceptor) closed to each other by the stem-loop structure of MB was constructed for monitoring VEGF mRNA. In the presence of VEGF mRNA, hybridization of mRNA and stem-loop structure disrupted the FL energy resonance transfer (FRET effect), and FL was quenched (Liu et al., 2019a), by which mRNA detection was realized.

In addition, by intrinsic peroxidase-like activity, MOFs could be used for glucose detection combination with FL production. The fluorescent biosensor, ultrathin two-dimensional Cu(bpy)_2_(OTf)_2_ nanosheets catalyzed non-fluorescent substrate thiamine (TH) to strong fluorescent thiochrome in the presence of H_2_O_2_ and thus were used for H_2_O_2_ analysis (Shi et al., 2019).

### 6.2 Chemiluminescence Detection

**FIGURE 13:**
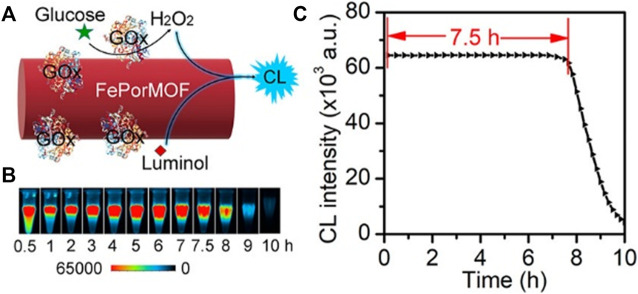
Chemiluminescence detection of nano-/bioenzymes based on FePorMOF/GOx nanoparticles. **(A)** Principle of chemiluminescence detection based on FePorMOF/GOx. **(B)** Chemiluminescence (CL) emission of nano-/bioenzymes. **(C)** Image analysis of FePorMOF/GOx with luminol and glucose. Reproduced with permission. Copyright 2020, *Analytical* *Chemistry*.

CL detection is based on light generated during chemical reactions. Luminol, *N*-(4-aminobutyl)-*N*-ethylisoluminol (ABEI), is the most commonly studied substrate for CL. Once oxidized in the presence of H_2_O_2_, it would show strong CL emission. Cu-MOF (Yang et al., 2021a) and Fe-MOF (Dang et al., 2020) (Figure 13) with intrinsic peroxidase activity have been demonstrated persistent CL through catalyzing the H_2_O_2_–luminol system. Additionally, β-CD-functionalized MOF (MOF-235/β-CD) with excellent catalytic performance exhibited more than 30-fold enhancement in CL intensity. Combined with GOx, this hybrid has been successfully applied for glucose detection (Mao et al., 2018b). Another ultrasensitive CL platform G4/MOFzyme, based on hemin-bridged MOFs, displayed about 100-fold higher catalytic activity to the H_2_O_2_–luminol system. When used in detecting acute myocardial infarction (AMI)-related miRNAs of human serum, their detection limit was lower than 1 fM. Moreover, when combined with a smartphone, point-of-care (POC) diagnosis of AMI would be realized, achieving higher sensitivity and longer duration for CL miRNA imaging (Mi et al., 2020).

### 6.3 Colorimetric Detection

Colorimetric detection based on MOFs is mainly dependent on catalytic MOFs. Catalytic MOFs catalyze their substrates such as 3,3′,5,5′-tetramethylbenzidine (TMB) in the presence of H_2_O_2_ to produce an intensive color reaction, which could be quantified through the colorimetric method. These catalytic MOFs could be obtained in three ways: peroxidase delivery (Zheng et al., 2020), nanozyme based on MOFs (Dong et al., 2017a; Chen et al., 2018a), and a combination of both (Zhao et al., 2019a; Xu et al., 2019b; Zhao et al., 2019b).

**FIGURE 14:**
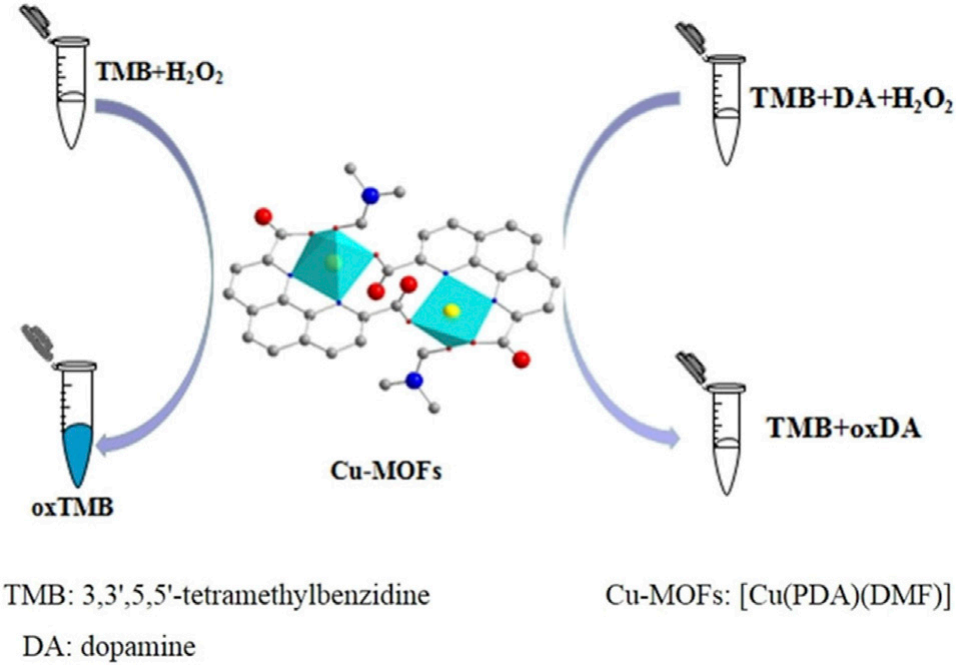
Schematic of the application of [Cu(PDA)(DMF)] with peroxidase-like activity in colorimetric dopamine detection. Reproduced with permission. Copyright 2019, *ACS Applied Materials & Interfaces*.

Wang et al. constructed a [Cu(PDA)(DMF)] MOF with peroxidase-like activity, which could catalyze colorless substrate TMB to a blue product. In contrast, this phenomenon could be inhibited by dopamine, which could be used for detecting dopamine in human urine and pharmaceutical samples through colorimetric methods (Wang et al., 2019b) (Figure 14). Besides, PCN-333(Fe), as a carrier of peroxidase (ficin), showed enhanced peroxidase activity towards TMB oxidation. When combining H_2_O_2_ generated from glucose oxidation, glucose detection was accomplished through a colorimetric method (Zheng et al., 2020).

## 7 Metal–Organic Framework Derivatives

In recent years, we also witnessed increasing applications of MOF derivatives in therapeutics and sensors. MOFs mainly exert roles such as sacrificial templates, or forming carbon nanostructures containing porphyrin-like metal centers (PMCS) after high-temperature calcination, or forming ultrasmall nanodots.

MOF derivatives based on MOF as sacrificial templates are for high loading anticancer drugs (Tang et al., 2017). Carbon nanostructure-containing PMCS from MOFs have excellent properties of sonosensitizer for SDT (Pan et al., 2018), photothermal conversion for PTT (Weng et al., 2020) and for CDT/PDT/PTT (Sui et al., 2021), and PAI-guided photothermal/photodynamic combination therapy (Yang et al., 2018a). Besides, ultrasmall porphyrinic MOF nanodots (MOF QDs) prepared from NMOFs could generate twofold effective ROS and thus benefit enhanced PDT. Interestingly, this ultrasmall nanostructure could be rapidly cleared from the kidney (Wang et al., 2019a).

Furthermore, with abundant atomically dispersed active metal ions and higher photothermal conversion ability, derivatives through pyrolysis have attracted increasing attention in the antibacterial application. For example, a single-atom nanozyme, formed after mesoporous silica (mSiO_2_)-protected pyrolysis of ZIF-8, with atomically dispersed active zinc atoms structure, has demonstrated peroxidase-like activity for antibacterial application (Xu et al., 2019a). Ag-doped MOF derivatives (C-Zn/Ag) exhibited a graphitic-like carbon framework after carbonization. Robust photothermal conversion abilities of graphitic-like structure and functions of Zn^2+^ and Ag^+^ ions made it possible for dual-functional sterilization (Yang et al., 2020).

## 8 Conclusion, Challenges, and Prospects

In this review, we conclude the most recent applications of MOFs and their composites in nanomedicine. First, various functionalities about MOFs are highlighted, including targeting modification, membrane biomimetics, responsiveness introduction, and other functionalities, aiming at constructing multifunctional MOFs for precise theranostics of diseases. Strategies of MOFs in the treatment of various diseases such as cancer and inflammatory diseases, imaging, sensors, and theranostics have been included without all applications included. Besides, we present applications of MOF derivatives in nanomedicine.

Although fruitful progress has been made in the treatment of diseases during the past few years, the majority of results are focused on cancer treatment, with fewer therapies on other diseases, including inflammatory diseases, antibacterials, and recent summary in regenerative medicine (Shyngys et al., 2021). Therefore, more intelligent therapies based on MOF for the abovementioned diseases are expected. For example, in the treatment for inflammatory diseases such as IBD, nanozymes based on MOF systems are increasingly being applied, in which MOFs are used as carriers or active components. In this therapy, inflammatory microenvironments such as ROS and immune cells were remodeled, and thus inflammation was alleviated. Gene therapy, a promising therapy modality, has been increasingly reported for its personalized treatment of diseases through specific genes involved. However, gene therapy based on MOFs is mainly focused on gene downregulation via siRNA, or CRISPR/Cas9 system, and there has been less coverage in the disease treatment. In addition, plasmid, another gene form, exhibits the capability of both upregulation and downregulation of specific genes but is rarely used, which may be caused by immune obstacles and targeting. MOFs, with superiority such as high loading and modifiability, have great potential in gene therapy. If challenges about MOF delivery of plasmids could be conquered, more smart nanostructures for personalized treatment would be fabricated.

Of course, there is still a long way to go before their clinical transformation, of which toxicity might be the most central factor. In most current reports, the toxicity of MOFs is evaluated through H&E staining of the main organs for a short term. Along with other crucial parameters including long-term toxicity, accumulation, and their toxicity of MOF components, the pharmacokinetics of MOFs is less frequently being reported, which also needs to be comprehensively considered. In addition, it also takes a longer time to evaluate their usability due to the large differences between species. Therefore, more efforts are needed in MOF platforms, from therapeutic modality exploitation for different diseases to overall toxicity evaluation before their clinical transformation.
