# Supplementary material for: Metal-Organic Frameworks and Their Composites Towards Biomedical Applications
Source: Front Mol Biosci. 2021 Dec 21;8:805228. doi: 10.3389/fmolb.2021.805228 (PMC8724581; doi:10.3389/fmolb.2021.805228)
Supplement: Supplementary file 1 [file Table1.docx]

**Supplementary Table 1. Examples of MOFs in cancer treatment.**

| **Therapy** | **MOF** | **Cargoes** | **Cell types** | **In vivo results** | **Functions of MOFs** | **Reference** |
| --- | --- | --- | --- | --- | --- | --- |
| Chemotherapy | ZIF-8 | CCM, CPT | HeLa | None | Nanocarrier | (Tiwari et al., 2017; Dong et al., 2019) |
|  | UiO-66-(SH)_2_ | 6-MP | SMMC-7721 | None | Nanocarrier;  GSH-responsive | (Gong et al., 2020) |
|  | ZIF-67/Fe_3_O_4_ | DOX | None | None | Nanocarrier | (Wang et al., 2018a) |
|  | ZIF-8-PAAS | DOX | HeLa | Yes | Nanocarrier | (Yan et al., 2017) |
|  | CMC@MOF-5 | 5-FU | HeLa | None | Nanocarrier;  Oral delivery | (Javanbakht et al., 2020) |
|  | ZIF-8 | DOX/VER | MCF-7 | Yes | Nanocarrier | (Zhang et al., 2017c) |
|  | ZIF-8 | DOX/Que | HepG2/ADR | Yes | Nanocarrier | (Li et al., 2019) |
|  | ZIF-90 | DOX/5-FU | None | None | Nanocarrier | (Zhang et al., 2017a) |
| Immunotherapy | ZIF-8 | CpG | RAW264.7 | None | Nanocarrier | (Zhang et al., 2017b) |
|  | Uio-AM | CpG | RAW264.7 | Yes | Nanocarrier | (Qi et al., 2019) |
|  | MIL-101-Fe-NH_2_ | OVA / CpG | DC2.4 | Yes | Nanocarrier | (Yang et al., 2018b) |
|  | ZIF-8 | NV |  | Yes | Nanocarrier | (Alsaiari et al., 2021) |
| Gene therapy | ZIF-8 | siRNA | SK-BR-3 | Yes | Nanocarrier | (Zhuang et al., 2020) |
|  | Se/Ru@MIL-101 | Pooled siRNA | MCF-7/T | Yes | Nanocarrier | (Chen et al., 2017b) |
|  | ZIF-8, ZIF-90 | Cas9/sgRNA for EGFP | HeLa/ MCF-7 | None | Nanocarrier | (Yang et al., 2019; Alyami et al., 2020) |
| PDT | ZIF-8@Ce6–HA | Ce6 | HepG2 cells | None | Nanocarrier | (Fu et al., 2020) |
|  | Sm-H_2_TCPP | None | MCF-7 | Yes | Photosensitizer | (Gao et al., 2019) |
|  | RC@TFC | RAP, Ce6, CAT | MDA-MB-231 | Yes | Nanocarrier | (Liu et al., 2019b) |
|  | MPEG2000-ZIF/PC composites (PMs) | PC, PPV | P3DCC | Yes | Nanocarrier | (Chen et al., 2021) |
|  | MOF-2 | None | HepG2 | Yes | Photosensitizer,  Decreased GSH | (Zhang et al., 2018c) |
|  | UMOF-TiO2 | None | MCF-7 | Yes | Photosensitizer,  Deeper penetration | (Shi et al., 2020) |
| CDT | HT@GOx-DMONs | GOx | MCF-7 | Yes | Gatekeeper,  Catalyst | (Wu et al., 2020) |
|  | ZIF-8@GOx/HRP | GOx/HRP | HeLa | Yes | Nanocarrier | (Bai et al., 2019) |
|  | Cu-Pd@MIL-101-PEG | None | HeLa,A549,4T1 | Yes | Nanocarrier | (Yang et al., 2021b) |
| SDT | P@HP | Ce6 | 4T1 cell | Yes | Nanocarrier | (An et al., 2020a) |
|  | D-MOF(Ti) | None | L929 and 4T1 cells | Yes | Sonosensitizer | (Liang et al., 2021) |
| Autophagy therapy | ZIF-8 | 3-MA (autophagy inhibitor) | HeLa cell | Yes | Nanocarrier | (Chen et al., 2018e) |
| Ferroptotic therapy | Cu-MOF (containing Fe) | Erastin(ferroptotic inducer) | MDA-MB-231 cells | Yes | Nanocarrier, sensitizers | (Xin et al., 2021) |
| Combination therapy |  |  |  |  |  |  |
| Chemotherapy/PDT | ZDZP@PP | DOX, PpIX | 4T1 | Yes | Nanocarrier | (Ren et al., 2020) |
| PDT/PTT | TPZ/Hf-TCPP/PEG  O_2_@UiO-66@ICG@RBC  Zn-TCPP | TPZ  O_2_, ICG  None | HeLa, 4T1  MCF-7  HeLa | Yes  Yes  None | Nanocarrier  Photosensitizer  Nanocarrier  Photosensitizer  Photothermal agent | (Liu et al., 2018)  (Gao et al., 2018a)  (Wang et al., 2019c) |

CCM: curcumin; CPT: camptothecin; 6-MP: 6-mercaptopurine; CMC: carboxymethylcellulose; 5-FU: 5-fluorouracil; VER, verapamil hydrochloride; Que, quercetin; OVA: ovalbumin; CpG: cytosine-phosphate-guanine oligonucleotide; NV: nivolumab; RAP: rapamycin; CAT: catalase; PC: phycocyanin; PPV: papaverine; GOx: glucose oxidase; HRP: horseradish peroxidase; PpIX: protoporphyrin IX; 3-MA: 3-methyladenine.
