# Supplementary material for: Metal-Organic Frameworks and Their Composites Towards Biomedical Applications
Source: Front Mol Biosci. 2021 Dec 21;8:805228. doi: 10.3389/fmolb.2021.805228 (PMC8724581; doi:10.3389/fmolb.2021.805228)
Supplement: Supplementary file 2 [file Table2.docx]

**Supplementary Table 2. Examples of MOFs in the treatment of inflammatory diseases.**

| **MOF** | **Cargoes** | **Diseases** | **In vivo results** | **Functions of MOFs** | **Reference** |
| --- | --- | --- | --- | --- | --- |
| γ-CD-MOF | DFNa | Paw edema | Yes | Carriers of anti-inflammatory drugs | (Abucafy et al., 2018) |
| MIL‑100(Fe) @HA | PCA | OA | Yes | Carriers of anti-inflammatory drugs, pH responsiveness | (Xiong et al., 2020) |
| Ce-MOF@PSS | 5-ASA | IBD | Yes | Carriers of anti-inflammatory drugs, active agents as ROS scavenger | (Yin et al., 2021) |
| ZIF-8 | CeO_2_ | Ischemic stroke | Yes | Carriers of nanozymes | (He et al., 2020) |
| MIL-47(V) | None | Ear inflammation; Colitis model | Yes | GPx-mimicking nanozymes | (Wu et al., 2021b) |
| Mg/HCOOH MOF | None | OA | None | Anti-inflammatory agents | (Li et al., 2020c) |
| PEI-ZIF-8 | None | Sepsis | Yes | cfDNA scavenger | (Liu et al., 2021a) |

DFNa: sodium diclofenac; PCA: protocatechuic acid; OA: osteoarthritis; 5-ASA: 5-amino salicylic acid; IBD: Inflammatory bowel disease; GPx: glutathione peroxidase.
