# Supplementary material for: Metal-Organic Frameworks and Their Composites Towards Biomedical Applications
Source: Front Mol Biosci. 2021 Dec 21;8:805228. doi: 10.3389/fmolb.2021.805228 (PMC8724581; doi:10.3389/fmolb.2021.805228)
Supplement: Supplementary file 3 [file Table3.DOCX]

**Supplementary Table 3. MOFs in imaging.**

| **Imaging types** | **Principles** | **Advantages** | **Active elements** | **MOFs** |
| --- | --- | --- | --- | --- |
| MR | Interaction between proton nuclei of samples and magnetic field | Noninvasive,  deeper penetration,  high resolution | Fe, Mn, Gd | Ga-MOF;  Fe-MOF:MIL family, Fe_3_O_4_-containing MOF;  Mn-MOF:PCN family(PCN-222,PCN-224); |
| CT | X-ray attenuation after tissues absorption | Deeper penetration,  high resolution | High atomic numbers (high-Z elements), like iodine, gold, bismuth, and gadolinium | iodinated MOF;  Zr- and Hf-based MOF; |
| PET | Monitoring metabolism processes by tracing signals from radioisotopes | Deep signal penetration, quantitative capacity,  high sensitivity | ^89^Zr, ^64^Cu, ^68^Ga | MOF based on ^89^Zr, ^64^Cu, ^68^Ga; |
| OI | Based on luminophore;  Including fluorescence, phosphorescence, bioluminescence | Real time monitoring,  high sensitivity | Luminophore | MOFs built from luminophore such as porphyrin-including MOFs;  Dyes-loaded MOF, such as RhB@ZIF-8;  MOFs composites |
| PA | Optoacoustic effect of tissue and contrast agent | Superb contrast,  super spatial resolution,  deeper penetration | Dyes with near infrared absorption, gold nanoparticles, carbon nanoparticles | MOFs containing active materials |

MR: magnetic resonance; CT: computed tomography; PET: positron emission tomography; OI: optical imaging; PA: photoacoustic imaging.
