# Supplementary material for: Metal-Organic Frameworks and Their Composites Towards Biomedical Applications
Source: Front Mol Biosci. 2021 Dec 21;8:805228. doi: 10.3389/fmolb.2021.805228 (PMC8724581; doi:10.3389/fmolb.2021.805228)
Supplement: Supplementary file 4 [file DataSheet1.DOCX]

November 18, 2021

Editorial Team

Frontiers in Molecular Biosciences

Dear Editor,

We would like to thank the editorial board and the reviewers for their thoughtful reviews of our manuscript entitled “**Metal-organic frameworks and their composites towards bio-medical applications**”. We have addressed the reviewers’ critiques and made corresponding changes in the manuscript where appropriate. We hope that our changes make this manuscript suitable for publication in *Frontiers in Molecular Biosciences*.

Sincerely,

Kangsheng Tu

**Reviewer 1#**

This review systematically summarizes the research progress of Metal-Organic Frameworks (MOFs) in biomedical therapy in recent five years and looks into the challenges and application prospects of MOFs in this field. The application of MOF-derivatives in the field of nanomedicine is also comprehensively summarized, which has a good reference value. Recently, Shyngys et al. published a mini-review article "Metal-Organic Framework (MOF)-Based Biomaterials for Tissue Engineering and Regenerative Medicine" (https://www.frontiersin.org/articles/10.3389/fbioe.2021.603608/full). The applications of MOF as biomaterials in tissue engineering and regenerative medicine are summarized. The above paper and this review are good complementary to the application of MOF as a biomaterial in biomedical research. It is recommended to add the above mini-review article as references where appropriate.

As suggested, we added this great reference into our revised manuscript on page 33, line 876.

**Reviewer 2#**

Owing to the unique features, metal organic frameworks (MOFs) and their composites have attracted increasing attention in biomedical applications. This review summarized the functionalization strategies of MOFs and their applications in the fields of therapeutics, imaging, theranostics and sensors in different disease models like inflammation and cancer. Finally, they had a prospective for MOFs’ further potential application. Scientifically, the paper is interesting, well performed and communicated in a professional manner. The text has a good flow. While the reviewer also has a few minor concerns as follows:

1. The author should provide high-resolution figure, like figure 11. And all figure legends should be in the same format, such as A, B. or (A), (B).

Thanks for your suggestion. As suggested, we re-downloaded the high-resolution image of original Figure 11, and added it to the revised manuscript as new Figure 14, on page 64. In addition, all figure legends have been revised in the same format.

2. In section 7. Conclusion, Challenges, and Prospects. I suggest the author divide this section and provide three parts for easily understand.

Thanks for your suggestion. As suggested, we re-organized this part into three parts on page 33-34, line 873 to line 899.

3. In section 7, the author highlighted more intelligent and precise MOF systems have been used in inflammation diseases such as IBD. I suggest the author summarize the related works in a table.

Thanks for your suggestion. We have summarized the applications of MOFs systems in inflammation diseases as follows:

**Table 2. Examples of MOFs in the treatment of inflammatory diseases.**

| **MOF** | **cargoes** | **Diseases** | **In vivo results** | **Function of MOF** | **Reference** |
| --- | --- | --- | --- | --- | --- |
| γ-CD-MOF | DFNa | Paw edema | Yes | Carriers of anti-inflammatory drugs | (Abucafy et al., 2018) |
| MIL‑100(Fe) @HA | PCA | OA | Yes | Carriers of anti-inflammatory drugs, pH responsiveness | (Xiong et al., 2020) |
| Ce-MOF@PSS | 5-ASA | IBD | Yes | Carriers of anti-inflammatory drugs, active agents as ROS scavenger | (Yin et al., 2021) |
| ZIF-8 | CeO_2_ | Ischemic stroke | Yes | Carriers of nanozymes | (He et al., 2020) |
| MIL-47(V) | None | Ear inflammation; Colitis model | Yes | GPx-mimicking nanozymes | (Wu et al., 2021) |
| Mg/HCOOH MOF | None | OA | None | Anti-inflammatory agents | (Li et al., 2020) |
| PEI-ZIF-8 | None | Sepsis | Yes | cfDNA scavenger | (Liu et al., 2021) |

DFNa: sodium diclofenac; PCA: protocatechuic acid; OA: osteoarthritis; 5-ASA: 5-amino salicylic acid; IBD: Inflammatory bowel disease; GPx: glutathione peroxidase.

In addition, we added a new report about MOF in treating for sepsis through scavenging the cfDNA produced by damaged cells both in text and table 2, on page 23, line 602 to 607.

4. The language should be polish, and spelling mistakes should be avoided in the revised manuscript.

Thanks for your suggestion. We carefully checked, polished and corrected the revised manuscript.

**Reviewer 3#**

In this review, the authors summarize that MOFs and their composites can be widely used in nanomedicine for drug delivery, therapeutics agents, imaging contrast agents, and sensor nanoreactors. The paper is both logical and detailed, and I suggest that the paper could be published in Frontiers in Molecular Biology after a minor revision.

1. In parts 2 and 4, there are only words, and it is suggested to add some illustrations.

Thanks for your suggestion. As suggested, we added new Figure 3, Figure 4, and Figure 9 into part 2.2.1, 2.3.1, 4.4, respectively.

**Figure 3.** Synthesis, application, and pharmacokinetics of erythrocyte membrane cloaked MOF, TGZ@eM. (A) Schematic illustration of TGZ@eM synthesis. (B) Application diagram of TGZ@eM in cancer treatment. (C) *In vivo* pharmacokinetics of TGZ@eM and TGZ. (D) Biodistribution of TGZ@eM or TGZ. (E) Blood glucose level of mice in time of day after injection with TGZ@eM. Reproduced with permission. Copyright 2018, ACS Nano.

**Figure 4.** Schematic illustration of HMS/MOFs pH-responsiveness. Reproduced with permission. Copyright 2018, ChemMedChem.

# Figure 9. Diagram of construction of multifunctional AuNC/Cam@MOF nanoprobe and its applications including FL imaging. Reproduced with permission. Copyright 2019, ANALYTICAL CHEMISTRY.

2. In section 3.1.4 phototherapy, PTT is mainly presented as a carrier, which is not summarized in detail in this review. Therefore, it is suggested that PDT and CDT be combined into a dynamic therapy part.

Thanks for your suggestion. We combined PDT and CDT into dynamic therapy. Meanwhile, we add sonodynamic therapy (SDT) into dynamic therapy. In addition, we re-organized the therapy including PTT, autophagy, ferroptotic therapy to part 3.1.5, on page 20, line 517 to line 530. Accordingly, Table 1 was also supplemented.

3. Grammar and tenses need to be checked again.

Thanks for your suggestion. We carefully checked, polished and corrected the revised manuscript.

**Reviewer 4#**

In this manuscript, authors summarized the applications of metal organic frameworks (MOFs) and their composites in bio-medicine in recent five years. Firstly, authors comprehensively introduced the types, characteristics and applications of MOF functionalities. Then they highlighted the application strategies of MOFs such as carriers and agents in therapeutics of different diseases, imaging, sensors and theranostics. Also, applications of MOFs derivatives were summarized. Lastly, challenges of MOFs in further applications and translations were described. This review will provide a good reference for further design about application of MOFs from functionalities to application strategies, so I agree to accept this review with minor revision.

Minor concerns and suggestions to improve this manuscript are as below:

Q1: In “Targeting modification” part, various targeting strategies were summarized and illustrated. Would it be better if comparative results between non-targeting and targeting strategies in each example were added in detail?

Thanks for your suggestion. As suggested, in the examples listed, we added the corresponding comparative results to text except for none. For example, we added the descriptions in “Part 2.2.1 HA-mediated targeting”, on page 4, from line 109 to line 112.

Q2: In “Conclusion, Challenges, and Prospects” part, the transitions between sentences are not smooth enough. For example, in line 857, transition from toxicity to other therapeutics is not logical enough, so language is suggested to be reorganized.

Thanks for your suggestion. We have corrected them. As suggested by Reviewer #2, we re-organized this part into three parts on page 33-34, line 873 to line 899.

Q3: Some sentences and words of this article are not so fluent or correct, for example, in line 209, the word spelling of “targeting” is incorrect, which need to be further modified.

Thanks for your suggestion. We carefully checked, polished and corrected the revised manuscript.
